# A CACTA-like transposon in the *Anthocyanidin synthase 1* (*Ans-1*) gene is responsible for apricot fruit colour in the raspberry (*Rubus idaeus*) cultivar ‘Varnes’

**DOI:** 10.1371/journal.pone.0318692

**Published:** 2025-02-03

**Authors:** Daniel James Sargent, Matteo Buti, Stefan Martens, Claudio Pugliesi, Kjersti Aaby, Dag Røen, Chandra Bhan Yadav, Felicidad Fernández Fernández, Muath Alsheikh, Jahn Davik, R. Jordan Price

**Affiliations:** 1 NIAB, Cambridge, United Kingdom; 2 Department of Agriculture, Food, Environment and Forestry, University of Florence, Florence, Italy; 3 Department of Food Quality and Nutrition, Fondazione Edmund Mach, Centro Ricerca e Innovazione, San Michele all’Adige, Trentino, Italy; 4 Department of Agriculture Food and Environment, University of Pisa, Pisa, Italy; 5 NOFIMA AS, Norwegian Institute of Food Fisheries and Aquaculture Research, Ås, Norway; 6 Njos Fruit and Berry Centre, Leikanger, Norway; 7 Graminor Breeding Ltd., Ridabu, Norway; 8 Department of Plant Sciences, Norwegian University of Life Sciences, Ås, Norway; 9 Division of Biotechnology and Plant Health, Norwegian Institute of Bioeconomy Research, Ås, Norway; Chiba Daigaku, JAPAN

## Abstract

Cultivated raspberries (*Rubus idaeus* L.) most commonly bear small, red, highly aromatic fruits. Their colour is derived predominantly from anthocyanins, water soluble polyphenolic pigments, but as well as red forms, there exist cultivars that display yellow- and apricot-coloured fruits. In this investigation, we used a multi-omics approach to elucidate the genetic basis of the apricot fruit colour in raspberry. Using metabolomics, we quantified anthocyanins in red and apricot raspberry fruits and demonstrated that, in contrast to red-fruited raspberries, fruits of the apricot cultivar ‘Varnes’ contain low concentrations of only a small number of anthocyanin compounds. By performing RNASeq, we revealed differential expression patterns in the apricot-fruited ‘Varnes’ for genes in the anthocyanin biosynthesis pathway and following whole genome sequencing using long-read Oxford Nanopore Technologies sequencing, we identified a CACTA-like transposable element (TE) in the second exon of the *Anthocyanidin synthase* (*Ans*) gene that caused a truncated predicted ANS protein. PCR confirmed the presence in heterozygous form of the transposon in an unrelated, red-fruited cultivar ‘Veten’, indicating apricot fruit colour is recessive to red and that it may be widespread in raspberry germplasm, potentially explaining why apricot forms appear at regular intervals in modern raspberry breeding populations.

## Introduction

Cultivated raspberries (*Rubus idaeus* L.) are economically important globally due to their sweet, delicate tasting berries, that have a pleasant flavour and aroma. The berries are usually red, small, weighing 5–10 grams commercially, and when ripe are rich in nutrients and bioactive compounds, including polyphenolics such as anthocyanins [1]. Anthocyanins are water soluble polyphenolic pigments responsible for the primary colouration in many plant species [2]. Their role in plant growth and development has yet to be fully elucidated, but they are recognised to have protective properties and are produced in response to biotic and abiotic stresses [3], as well as being the principal component of the characteristic colour of commercial raspberry fruits [1, 4].

There exist raspberry cultivars that display fruits that are yellow and apricot in colour, rather than red. These phenotypes have been historically shown to be attributable to mutations in a small number of major genes, *T* which was shown to give yellow fruits in homozygous recessive form [5], and *P* which gave apricot fruit colour in combination with *T*, with which it was reported to interact epistatically [6]. Later, a further gene *R* was proposed to be the one controlling the accumulation of rhamnose-containing anthocyanin pigments in raspberry fruits [7].

Several key enzymes form the anthocyanin biosynthesis pathway in plants, including chalcone synthase (CHS), chalcone isomerase (CHI), flavanone-3-hydroxylase (F3H), dihydroflavanol-4-reductase (DFR), anthocyanidin synthase (ANS) and UDP 3-O-glycosyltransferase (UGT), resulting in the synthesis of pelargonidin 3-O-glycosides. The activity of flavonoid 3′-hydroxylase (F3′H) is necessary to channel the metabolic flux into the direction of the main anthocyanins in raspberry, resulting in the production of different cyanidin 3-O-glycosides [8]. The structural genes of the anthocyanin biosynthetic pathway function under control of a regulatory complex, called the MYB-bHLH-WD40 (MBW) complex, consisting of MYB, basic helix-loop-helix (bHLH) and WD40 repeat families [9].

The genes involved in the anthocyanin biosynthesis pathway have been well-characterised in fruiting plants species such as grapevine and those closely-related to raspberry such as apple [10] and strawberry [11–13]. The genetic causes of colour mutants of flowers and fruits have been attributed to mutations in the anthocyanin biosynthesis pathway genes in many plant species where, in some cases, the presence of transposable elements (TEs) have been shown to cause alterations in gene function [14–16].

Among TEs, CACTA TEs represent one of the most widespread superfamilies of class II transposons. CACTA TEs are found in most genomes, from algae [17] to vascular plants [18–21], and animals [22]. CACTA elements can rearrange host genomes by altering the structure and regulation of individual genes through various processes, such as transposition, insertion, excision, chromosome breakage, and ectopic recombination [23, 24]. In maize, the classical *Enhancer/Suppressor mutator* (*En/Spm*) TE was the first observed CACTA element, independently identified by Peterson [25] and McClintock [26] and molecularly characterized by Pereira *et al*. [18].

The genetic basis of gene *T* giving rise to yellow-fruited raspberry varieties was studied in detail [27]. The authors demonstrated that a five base-pair insertion in the *Anthocyanidin synthase* (*Ans*) gene in the cultivar ‘Anne’ was responsible for a premature stop codon in the predicted ANS amino acid sequence, leading to a downregulation of *Ans* transcription via nonsense-mediated mRNA decay and a loss of ANS function. They showed that the berries, and indeed all other tissues of ‘Anne’ were devoid of anthocyanins, consistent with the gene *T* reported by [5], and provided evidence that the mutation they discovered in the *Ans* gene was responsible for the loss of anthocyanin production as the mutant allele was unable to restore anthocyanin production in *Arabidopsis* transgenic lines.

In addition to ‘yellow’ raspberry cultivars, there are many examples of the ‘apricot’ forms previously attributed to genes *T* and *P* [5], which produce fruits with varying degrees of intensity of colour. The colouration produces attractive berries and as such, some of these forms have been developed into commercial varieties. Metabolomic analysis of these variants has not previously been performed, so it is unknown what causes the ‘apricot’ colouration of the fruit, but overripe fruits of these cultivars have deeper pigmentation suggesting that, unlike the ‘yellow’ variants, anthocyanins are produced in at least some of these cultivars, but at much lower levels than in red forms. A greater understanding of the genetic control of anthocyanin production in raspberry would be useful for breeding, where selection of particular colour variants is desirable, and for selecting for breeding lines enriched with health-promoting antioxidants.

Genomics research has progressed rapidly in recent years for raspberry and several chromosome-length genome sequences for the species have been published and their sequences made publicly available to the research community [28–30]. Chromosome-scale assemblies greatly facilitate the investigation of gene structure and function and the elucidation of genetic factors causing economically important phenotypes. Here we present the findings of multi-omics (metabolomics, transcriptomics and genomics) studies to elucidate the genetic mechanisms for the characteristic ‘apricot’ pigmentation of berries of the raspberry cultivar ‘Varnes’ and, in doing so, identify a novel mutation in the anthocyanidin synthase gene caused by a putative non-autonomous CACTA-like TE present at the same location of the *Ans* gene as the loss of function insertion previously characterised in the cultivar ‘Anne’ [27].

## Materials and methods

### Plant material and growth

Four raspberry cultivars were investigated in this experiment, the red-fruited cultivars ‘Anitra’, ‘Glen Ample’ and ‘Veten’, and the apricot-fruited ‘Varnes’. Plant material, clonally propagated from root blocks, was grown in soil on raised beds covered with woven plastic mulch and supplemented with drip fertigation. The plants were grown at a distance of 0.5 m within rows and 3.5 m between rows. All plants used in this study were grown as part of the breeding program at Njøs Fruit and Berry Centre in Leikanger, Norway, at 61°13’ N and 6°47’ E, and as such, no permits were necessary to acquire material. The flowers were pollinated naturally using Bumblebees.

### Fruit sample preparation

Developing fruits of the raspberry cultivars ‘Anitra’, ‘Glen Ample’, ‘Varnes’ and ‘Veten’ were collected at three maturity stages; (1) unripe (at 25 days post-anthesis), (2) turning (at 30 days post anthesis), and (3) fully mature (35 days post anthesis). Fruits of ‘Anitra’, ‘Glen Ample’ and ‘Veten’ are various shades of red when fully ripened, whilst the fruits of ‘Varnes’ remain yellow/orange and never turn fully red. Fruit samples from each cultivar at each maturity stage were divided into 3 biological replicates, each with 15 berries with stage-representative characteristics [31]. Each fruit in the 36 samples (4 cultivars × 3 maturity stages × 3 replicates) were divided into two halves, with one half used for RNA extraction, and the other used for metabolomic analysis. The berries were frozen in liquid nitrogen and kept at -80°C until extraction and analysis.

### Extraction of phenolic compounds from raspberry fruits

The frozen fruit samples (10–60 g) were freeze-dried for four days (Gamma 1–16, Christ GmbH, Osterode am Harz, Germany), then milled in a mortar with pestle and stored in the dark at 6°C prior to extraction. Milled freeze-dried samples (0.400 g) were extracted with 70% acetone (5 ml) by sonication for 10 min. After centrifugation (1500 x *g* for 10 min at 4°C, Heraus Multifuge 4KR, Kendro Laboratory Products GmbH, Hanau, Germany), the supernatant was collected, and the insoluble plant material was re-extracted with 70% acetone (5 ml). Acetone was removed from the pooled extracts by nitrogen flow at 37°C (Sample concentrator, Techne, Stone, Staffordshire, UK). The volume of the extracts was made up to 5 ml with water and were stored at—80°C until analysis.

### Analysis of phenolic compounds with HPLC-DAD-MSn

The extracts were filtered through HA 0.45 μm filters (Millipore Corp., Billerica, MA, USA), and injected (5 μL) on an Agilent 1100 series HPLC system (Agilent Technologies, Waldbronn, Germany) equipped with an auto-sampler cooled to 4°C, a diode array detector, and a MSD XCT ion trap mass spectrometer fitted with an electrospray ionization interface as previously described [1]. Chromatographic separation was performed on a Synergi 4 μm MAX RP C12 column (250 mm × 2.0 mm i.d.) equipped with a 5 μm C12 guard column (4.0 mm × 2.0 mm i.d.), both from Phenomenex (Torrance, CA, USA), with mobile phases consisting of A; formic acid/water (2/98% v/v) and B; acetonitrile. The phenolic compounds were identified based on their UV–vis spectra (220–600 nm), mass spectra and retention times relative to external standards and comparison with previous results [32, 33]. The phenolic compounds were classified based on their characteristic UV–vis spectra and quantified by external standards. Anthocyanins were quantified against cyanidin-3-sophoroside at 520 nm. All results were expressed as μg g^-1^ of dry weight (DW).

### RNA extraction, library construction, and RNA sequencing

Total RNA was extracted from all fruit tissue samples using the RNeasy Plant Mini Kit (Qiagen, Oslo, Norway) following the manufacturer’s instructions. The concentration and purity of the resultant RNA was measured using a QIAxpert spectrophotometer (Qiagen, Oslo, Norway) and the integrity of the RNA was determined using a Qubit 4.0 fluorimeter (Thermo Fisher Scientific, Dartford, UK). Samples with an RNA integrity number (RIN) value above 7.0 were submitted for subsequent library preparation and sequencing. Library preparation was performed for the 36 fruit samples using the NEB Next^®^ ultra-RNA library prep kit (Biolabs, Inc., Beijing, China) and 125-bp paired-end Illumina sequencing was performed by Norwegian Sequencing Centre (Oslo University Hospital, Norway) using the HiSeq2500 platform (Illumina Inc. San Diego, CA, USA) to yield a minimum of 12 GB of data per sample.

### Global differential gene expression analysis

The quality of the RNASeq libraries was assessed using FastQC v0.11.9 (Andrews 2010), whilst poor quality reads and TruSeq adapters sequences were filtered out with Trimmomatic 0.39 [34] (parameters: ILLUMINACLIP:adapters.fa:2:30:10 LEADING:3 TRAILING:3 SLIDINGWINDOW:4:15 MINLEN:36). Filtered reads of all the libraries were mapped to the ‘Malling Jewel’ genome sequence [30] using HiSat2 v2.2.1 [35] with default parameters. Reads mapping to each predicted transcript were counted with featureCounts v2.0.3 [36] using “exon” as the feature and “transcript” as the attribute, eliminating chimeric counts. Raw counts were normalized based on read number of each individual library, and non-expressed or poorly expressed transcripts were filtered out; a transcript was considered ‘active’ if CPM (counts per million mapped reads) was ≥1 in at least two libraries. Multi-dimensional scaling (MDS) plot for RNA libraries normalized counts were visualized for the three experiments while using the ‘plotMDS’ command of Bioconductor EdgeR v3.38.1 [37]. Differential expression (DE) analyses were carried out for two pairwise comparisons for each of the four cultivars (‘turning’ vs. ‘unripe’ and ‘mature’ vs. ‘unripe’) using Bioconductor EdgeR v3.38.1 [37] employing the likelihood test. A transcript was considered differentially expressed in a pairwise comparison if the false discovery rate (FDR) was lower than 0.05 and log_2_FC (fold change) was lower than -2 or higher than 2.

### Differential expression of anthocyanin biosynthesis pathway genes in developing fruit tissue

Annotated full length sequences of the anthocyanin biosynthesis pathway genes phenylalanine lyase (*Pal*), chalcone synthase (*Chs*), chalcone isomerase (*Chi*), flavanone-β3-hydroxylase (*F3h*), dihydroflavanol-4-reductase (*Dfr*), and anthocyanadin synthase (*Ans*) of closely related species including *Fragaria*, *Malus* and *Prunus*, were retrieved from NCBI and used as queries to identify the corresponding genomic locations of homologues in the *R*. *idaeus* ‘Malling Jewel’ genome sequence [30] using BLASTn. The resultant corresponding coding domain sequence (CDS) of the ‘Malling Jewel’ gene predictions [30] were confirmed based on the RNASeq reads mapped from each of the raspberry cultivars studied. SAMtools v1.17 [38] was used to calculate the number of reads mapping to each nucleotide of ‘Malling Jewel’ anthocyanin biosynthesis gene homologues for each RNA library starting from.bam alignment files obtained with HiSat2 v2.2.1 [35]. Subsequently, the average number of mapped reads of the nine RNA libraries (3 fruit stages × 3 replicates) for each nucleotide were calculated and plotted for each cultivar using ggplot2 R package [39]. Gene expression levels of the anthocyanin biosynthesis pathway genes for each cultivar and each stage of maturity were calculated as transcripts per million mapped reads (TPM). Relationships between gene expression and anthocyanin biosynthesis in each cultivar investigated was explored using WGCNA [40] running default parameters.

### High molecular weight genomic DNA extraction and sequencing of the apricot raspberry cultivar ‘Varnes’ genome

In order to investigate the anthocyanin genes further in ‘Varnes’, high molecular weight DNA was extracted for long read sequencing from fresh, young leaf material collected from a single plant of the cultivar and submitted for sequencing using the Oxford Nanopore and Illumina sequencing platforms. The DNA extraction protocol of Schalamun *et al*. [41] was used with minor modifications; Sera-Mag SpeedBead magnetic carboxylate modified particles were used, 1% PVP-10 and 1% PVP-40 along with 1% Sodium metabisulfite was added to the tissue immediately before grinding under liquid nitrogen with a mortar and pestle, and the chloroform:isoamyl alcohol steps were performed twice. Multiple extractions from tissue of the same plant were performed and combined after precipitation. Long-read sequencing was performed using the Oxford Nanopore platform. Sequencing libraries were prepared using the SQK-LSK114 Ligation Sequencing Kit (Oxford Nanopore Technologies) from approximately 1 μg of high molecular weight genomic DNA, following the manufacturer’s protocol. The resultant long-read libraries were sequenced on a single Oxford Nanopore R10 Flow cell with Q20+ chemistry using the PromethION platform (Oxford Nanopore Technologies) set to high accuracy base calling by Novogene (Cambridge UK). Additionally, a PCR free short read Illumina sequencing library was prepared for ‘Varnes’ using an insert size of 350 bp. The library was sequenced with 150 bp paired-end reads on the Illumina NovaSeq X Plus platform at Novogene UK (Cambridge, UK).

### Structural variant analysis

The long-read sequencing data of the ‘Varnes’ genome were quality controlled using NanoPlot v1.30.1 [42] and sequencing adapters were trimmed using Porechop v0.2.4 (https://github.com/rrwick/Porechop) using default parameters. Following trimming, reads were filtered with a minimum Q score of 17 and a minimum read length of 1 kb using Filtlong v0.2.1. This high-quality reads set was aligned to the ‘Malling Jewel’ genome sequence using LRA v1.3.7.1 [43]. The resulting SAM file was converted to BAM format, sorted and indexed using SAMtools v1.17 [38]. Structural variants were called using SVIM v1.4.2 [44] using the ‘alignment’ command with default parameters. Variants were filtered using BCFtools v1.17 [45] using the following expression ’QUAL>40 && SUPPORT>10 && FILTER = "PASS‴.

### ‘Varnes’ genome assembly

Trimmed reads were filtered with a minimum Q score of 12 and a minimum read length of 10 kb using Filtlong v0.2.1 (https://github.com/rrwick/Filtlong). Long reads were assembled using the filtered long-read dataset using NECAT v0.0.1_update20200803 [46] using a coverage of 80× and specifying a genome size of 270 Mb, reflecting the approximate genome size of the other assembled raspberry genomes [30]. Following assembly, Purge_Dups v1.0.1 [47] was used with default settings to remove heterozygous contigs and overlapping heterozygous contigs. LongStitch [48] was used with default parameters to correct and scaffold the ‘Varnes’ assembly, and error correction and polishing was subsequently performed following long read alignment with Minimap2 v2.17-r941 [49] with Racon v1.4.20 [50] and Medaka v1.11.3 (https://github.com/nanoporetech/medaka) using the r1041_e82_400bps_sup_g615.

The Illumina paired-end reads were quality controlled with FastQC v0.11.9 (https://www.bioinformatics.babraham.ac.uk/projects/fastqc/), and adapters and low-quality regions were trimmed using Trimmomatic v0.39 [34]. Short reads were aligned to the purged and corrected long-read ‘Varnes’ assembly using Bowtie2 v2.4.4 [51] and SAMtools v1.17 [38] and three iterations of polishing were performed using Pilon v1.24 [52]. The processed assembly was used as a query against the NCBI mitochondria and chloroplast databases and organellar DNA contigs were removed from the contig set. A custom repeat library was generated using RepeatModeler v2.0.5 [53]. Using this library, RepeatMasker v4.1.6 [54] was used to annotate and softmask the repeat content of the ‘Varnes’ genome assembly, and any small contigs containing only repetitive DNA were removed from the assembly. The remaining contigs were ordered, orientated and scaffolded into pseudochromosomes using RagTag v2.1.0 [55] with the ‘Malling Jewel’ reference genome sequence. Assembly statistics for the polished genome were generated using a custom Python script, and single copy ortholog analysis was performed with BUSCO v5.2.2 [56], using the eudicots_odb10 database.

### ‘Varnes’ genome annotation and anthocyanin biosynthesis gene analysis

The ‘Varnes’ RNASeq reads described above were aligned to the soft-masked genome sequence with default settings using HiSat2 v2.2.1 [35] and gene prediction was performed using BRAKER3 [57] using the eudicots orthoDB database and the published *R*. *idaeus* proteomes as evidence. Global differential gene expression analysis was performed as described above using the ‘Varnes’ genome sequence and gene predictions, following which the genome regions containing the anthocyanin biosynthesis genes were identified using BLASTn homology analysis, and the expression of these genes in the ‘Varnes’ genome was analysed. Plots of the read mapping performed with HiSat2 v2.2.1 [35] to the anthocyanin biosynthesis gene homologues of ‘Varnes’ were produced, along with plots of gene expression levels (expressed as counts per million mapped reads; CPM) of the anthocyanin biosynthesis pathway genes at each stage of maturity. Transcription levels (expressed as transcripts per million mapped reads; TPM) of the ‘Varnes’ anthocyanin biosynthesis pathway genes at each stage of maturity were calculated for each nucleotide following the protocol described above for the ‘Malling Jewel’ genome using SAMtools v1.17 [38] and plotted with ggplots2 [39].

The *Ans* gene locus was further compared between ‘Varnes’ and ‘Malling Jewel’ by performing a MAFFT alignment in Geneious Prime v2023.0.4 (https://www.geneious.com). The results were visualised using NGenomeSyn v1.41 [58].

### Functional annotation of the Anthocyanidin synthase gene locus in ‘Varnes’ and ‘Malling Jewel’

Analysis of protein domains in the anthocyanidin synthase gene in ‘Malling Jewel’ and ‘Varnes’ was performed using Expasy [59] running default parameters. Positions of the catalytic sites in the full-length *Ans* gene of ‘Malling Jewel’ were determined by comparison to ANS proteins in the EBI alpha-fold database (alphafold.ebi.ac.uk) and comparison to the *Ans* gene from ‘Varnes’ was done by aligning the predicted protein sequences in MAFTT (https://www.ebi.ac.uk/jdispatcher/msa/mafft).

### Characterisation of the ‘Varnes’ Ans insert sequence

Whole genome *de-novo* transposable element annotation was performed on the ‘Varnes’ genome assembly using EDTA v2.2.1 [60] with default settings. The nucleotides representing the terminal inverted repeat (TIR) sequences (i.e. first 10 bp and last 10bp) for all ‘CACTA_TIR_transposon’ annotations were extracted and the 5’ and 3’ sequences concatenated. A multiple sequence alignment of these sequences, together with the corresponding sequences from the ‘Varnes’ *Ans* gene insert, was performed using Clustal Omega v1.2.2 [61]. A phylogenetic tree was generated using FastTree v.2.1.12 [62] and visualised using iTOL v6 [63].

### Confirmation of Ans alleles in ‘Anitra’, ‘Glen Ample’, ‘Varnes’ and ‘Veten’

Young, newly emerging leaf tissue of the four raspberry cultivars, ‘Anitra’, ‘Glen Ample’, ‘Varnes’ and ‘Veten’, studied in this investigation were freeze dried and ground to a fine powder, following which DNA was extracted using the DNeasy Plant Miniprep kit (Qiagen) according to the manufacturer’s recommendations. The DNA was resuspended in 200 μl of AE elution buffer and the samples were assessed for purity and quantified using a Nanodrop spectrophotometer (ThermoFisher Scientific) and a Qubit 4.0 fluorometer (ThermoFisher Scientific).

Primers were designed from the *Ans* gene of the ‘Varnes’ and ‘Malling Jewel’ genome sequence to amplify the full *Ans* gene region in the four cultivars. Additionally, primers were designed spanning the *Ans*/CACTA-like insertion site to amplify an allele-specific product for the allele containing the CACTA-like TE. Primers were designed using the software PRIMER3 [64]. The criteria for design were a Tm of 55–65°C (optimum 60°C), a primer length of 20–24 bp (optimum 22 bp) and a 2 bp GC-clamp at the 5’ end. All PCR reactions were performed in a final volume of 25 μL containing 2 ng genomic DNA, 1 × PCR buffer (NEB), 1.5 mM MgCl_2_, 0.2 mM of each dNTP, 0.2 μM of each primer, and 0.5 U of Phusion high-fidelity DNA Polymerase (NEB). The following PCR protocol was used: denaturation (98°C for 30 s), followed by 40 cycles of: 98°C for 10s, 58°C for 30s and 72°C for 1 min, followed by a final elongation step of 72°C for 10 min. The resultant PCR products were electrophoresed at 80V on 1.5% TAE agarose gel, stained with GelRed^®^ Nucleic Acid Gel Stain (biotium) and visualized over UV light.

The amplified PCR products were eluted from the gel and purified using the Wizard(R) SV Gel and PCR Clean-up kit (Promega Corporation) and cloned into pGEMT Easy Vector (Promega Corporation, Cat. No. A1360) following the manufacturer’s instructions. The ligation mixture was transformed into the DH5α strain of *E*. *coli* (NEB, Cat. No. C2987) following manufacturer’s protocol. Transformed *E*. *coli* colonies were confirmed through colony PCR using the *Ans* gene specific primers described above. Plasmid DNA was extracted using the Wizard^®^ Plus SV Miniprep kit (Promega Corporation, Cat. No. A1330) and positive clones were subjected to restriction analysis to further confirm the expected size of the cloned PCR products. Plasmid DNA for each of the full-length *Ans* genes cloned was sequenced using Sanger sequencing by IDT (Leuven, Belgium).

## Results

### Fruit ripening in raspberry cultivars ‘Anitra’, ‘Glen Ample’, ‘Varnes’ and ‘Veten’

**Fig 1:**
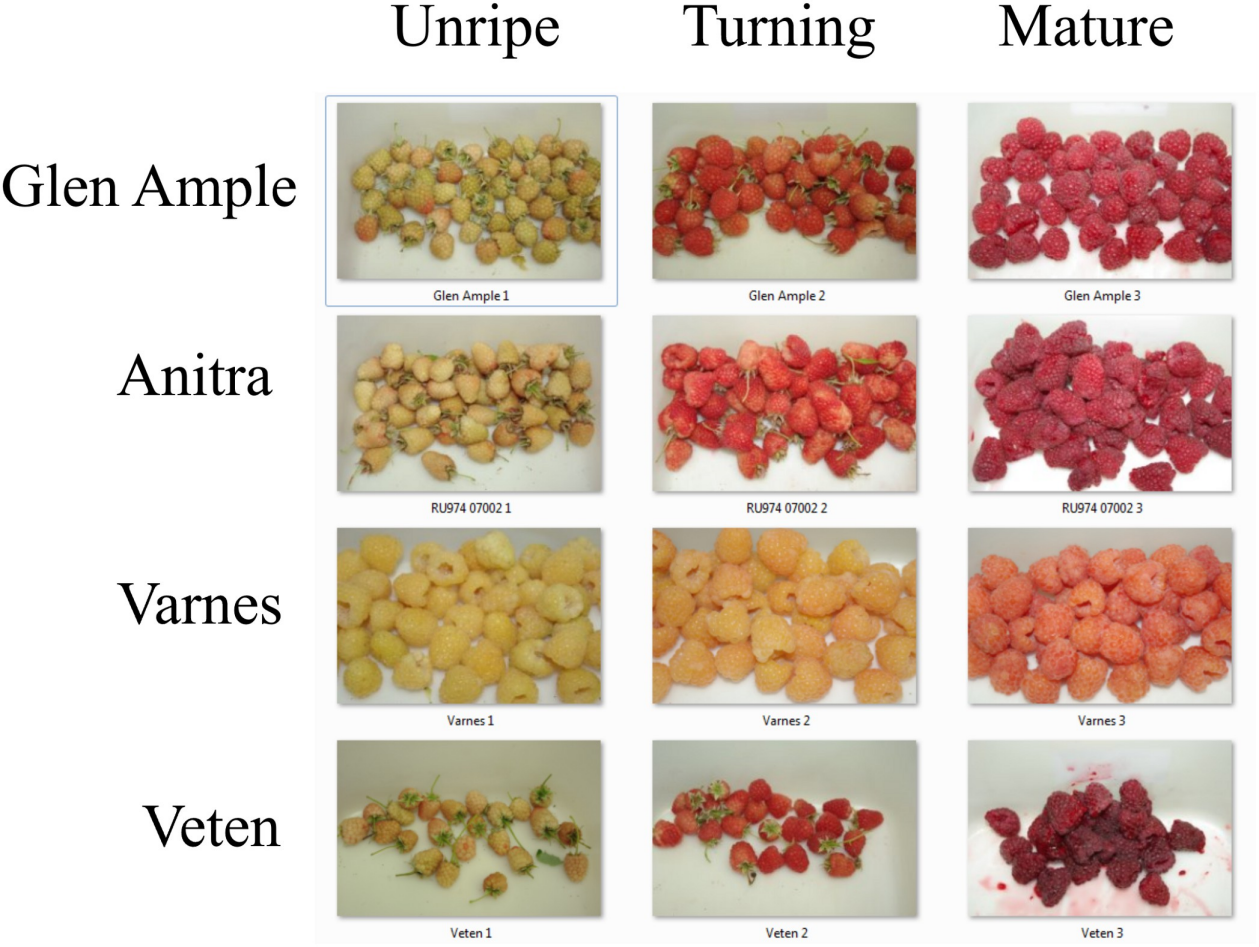
Fruit samples of the red-fruited raspberry cultivars ‘Anitra’, Glen Ample’ and ‘Veten’ and the apricot-fruited cultivar ‘Varnes’ at three stages of development: (1) unripe (at 25 days post-anthesis), (2) turning (at 30 days post anthesis), and (3) fully mature (35 days post anthesis).

The developing fruit harvested at three developmental stages, ‘unripe’, ‘turning’ and ‘mature’ from the four raspberry cultivars investigated, ‘Anitra’, Glen Ample’ and ‘Veten’ and ‘Varnes’ used for metabolomic and gene expression analysis are shown in Fig 1. The fruit of ‘Varnes’ shows a clear lack of red pigmentation at the ‘turning’ and ‘mature’ stages, with fruit displaying a yellow colour in the ‘unripe’ and ‘turning’ stages, turning apricot in colour when fully mature, in contrast to the red colouration of the other three cultivars (Fig 1).

### Analysis of polyphenol compounds in developing raspberry fruit

The mean concentrations (μg g^-1^ DW) of anthocyanins produced by fruit samples of four raspberry cultivars at the three stages of fruit development (unripe, turning and mature fruit) are given in Table 1. In mature fruit samples total anthocyanin content ranged from 232 μg g^-1^ DW in ‘Varnes’ to 8071 μg g^-1^ DW in ‘Veten’, indicating all cultivars produced anthocyanins but that ‘Varnes’ produced significantly lower concentrations of anthocyanins than the other cultivars. The DW of the raspberry samples were on average 15.3% (Table 1). When calculated on a fresh weight basis (FW), the total anthocyanin concentrations of mature fruits of the three red-coloured cultivars were 63–123 mg 100 g^-1^ FW. Whilst ten anthocyanins, with cyanidin-3-sophoroside, cyanidin-3-(2G-glucolsyrutinoside), cyanidin-3-glucoside and cyanidin-3-rutinoside as the major compounds could be detected and were quantified in the three red-fruited cultivars, only cyanidin-3-sophoroside, and cyanidin-3-glucoside were detected in fruit samples of ‘Varnes’ (Table 1). Principle components analysis of the major anthocyanin compounds (S1 Fig) showed a clear separation of the ‘mature’ fruit samples in the three red-fruited cultivars ‘Anitra’, ‘Glen Ample’ and ‘Veten’ along the first and second principal components, whilst the ‘mature’ samples of ‘Varnes’ clustered with the ‘unripe’ and ‘turning’ samples from all four cultivars, highlighting the very different profile of anthocyanin production during ripening in that cultivar.

**Table 1:** Concentrations (μg g^−1^ DW) of ten anthocyanin compounds analysed in raspberry (*Rubus idaeus*) fruit samples at three stages of maturity, ‘unripe’, ‘turning’, and ‘mature’ in four raspberry cultivars, ‘Anitra’, ‘Glen Ample’ and ‘Veten’ (red-fruited) and ‘Varnes’ (apricot-fruited).

Genotype	Developmental stage	Dry matter	cyanidin-3,5-diglucoside	cyanidin-3-sophoroside	cyanidin-3-(2G-glucosylrutinoside)	cyanidin-3-glucoside	pelargonidin-3-sophoroside
Anitra	Unripe	15.8 ± 0.3	0	0	0	38.7 ± 4.7	0
	Turning	14.6 ± 0.3	0	520 ± 48.7	296.0 ± 39.1	157.3 ± 8.3	14.7 ± 2.0
	Mature	14.5 ± 0.3	0	2633.7 ± 65.7	2330.7 ± 64.6	586.3 ± 21.9	116.3 ± 6.2
Glen Ample	Unripe	15.8 ± 0.3	0	14.0 ± 1.0	0	75.7 ± 1.7	0
	Turning	15.3 ± 0.2	0	326.3 ± 10.7	175.3 ± 8.9	217.7 ± 17.3	0
	Mature	15.0 ± 0.1	57.8 ± 1.5	1057.3 ± 4.9	1127.3 ± 11.9	940.7 ± 49.9	29.7 ± 3.0
Varnes	Unripe	15.7 ± 0.1	0	0	0	0	0
	Turning	16.1 ± 0.3	0	27.3 ± 1.2	0	0	0
	Mature	16.2 ± 0.4	0	159.0 ± 5.0	0	72.5 ± 3.5	0
Veten	Unripe	14.0 ± 0.1	0	0	0	93.7 ± 3.8	0
	Turning	15.2 ± 0.2	0	278.7 ± 73.7	107 ± 25.1	356.3 ± 62.4	0
	Mature	15.2 ± 0.0	74.9 ± 5.2	1928.0 ± 119.0	1126.7 ± 88.2	2827.3 ± 321.4	82.3 ± 6.4
Genotype	Developmental stage	cyanidin-3-xylosylrutinoside	cyanidin-3-rutinoside	pelargonidin-3-(2G-glucosylrutinoside)	pelargonidin-3-glucoside	pelargonidin-3-rutinoside	Total anthocyanins
Anitra	Unripe	0	0	0	0	0	38.7 ± 4.7
	Turning	0	87.33 ± 7.1	18.0 ± 4.0	0	0	1094.0 ± 108.9
	Mature	30.3 ± 2.4	443.3 ± 13.4	180.0 ± 14.6	25.3 ± 2.2	19.0 ± 1.5	6364.7 ± 173.5
Glen Ample	Unripe	0	37.7 ± 0.7	0	0	0	127.7 ± 1.8
	Turning	0	109.3 ± 3.8	0	0	0	829.0 ± 28.2
	Mature	45.7 ± 3.5	849.7 ± 41.6	51.3 ± 5.2	25.3 ± 3.7	33.3 ± 4.2	4217.7 ± 117.1
Varnes	Unripe	0	0	0	0	0	0
	Turning	0	0	0	0	0	27.3 ± 1.2
	Mature	0	0	0	0	0	232.0 ± 8.0
Veten	Unripe	0	35.7 ± 2.3	0	0	0	129.3 ± 5.8
	Turning	0	151.0 ± 23.2	0	0	0	893.7± 183.9
	Mature	45.0 ± 3.8	1687.0 ± 192.4	73.7 ± 6.0	135.7 ± 26.9	91.7 ± 15.9	8071.3 ± 622.3

### Differential gene expression in developing raspberry fruit

Raw data from the 36 libraries were deposited in the ArrayExpress repository at EMBL-EBI (www.ebi.ac.uk/arrayexpress) under the accession number E-MTAB-14165. Between 92.1–95.7% of filtered reads from the 36 RNA libraries mapped to the *Rubus idaeus* ‘Malling Jewel’ reference genome sequence. After the non-expressed and poorly expressed transcripts were removed, the raw counts of 19,010 genes identified as ‘active’ were normalized according to the dimensions of each library. Eight differential expression (DE) analyses were carried out, comparing for each cultivar the ‘turning’ and ‘fully mature’ fruit transcriptomes against the ‘unripe’ fruit transcriptome. The number of differentially expressed transcripts (DETs) was greater in ‘fully mature’ samples than in ‘turning’ samples for all cultivars, compared to ‘unripe’ fruits, whilst the number of down-regulated and the number of up-regulated transcripts was similar between ‘turning’ and ‘unripe’ samples, there were significantly more down-regulated transcripts than up-regulated transcripts in the ‘mature’ versus ‘unripe’ comparisons (Table 2).

**Table 2:** Differential expression analysis statistics. Numbers of down-regulated and up-regulated differentially expressed transcripts for ‘turning’ vs. ‘unripe’ (Tu vs. Un) and ‘mature’ vs. ‘unripe’ (Ma vs. Un) pairwise comparisons revealed in developing fruit of the raspberry cultivars ‘Anitra’, ‘Glen Ample’, ‘Varnes’ and ‘Veten’.

Cultivar	Pairwise Comparison	Down-regulated	Up-regulated
Anitra	Turning vs. Unripe	242	198
	Mature vs. Unripe	3,326	1,567
Glen Ample	Turning vs. Unripe	226	293
	Mature vs. Unripe	3,311	1,498
Varnes	Turning vs. Unripe	329	326
	Mature vs. Unripe	2,860	1,619
Veten	Turning vs. Unripe	290	373
	Mature vs. Unripe	3,067	1,747

The similarities between the gene expression data of each replicate of the four cultivars studied at the three investigated stages of fruit maturity were scrutinised in relation to the content of anthocyanins produced by each sample using WGCNA (S2 Fig). The data revealed that gene expression data from replicates taken at the same maturity stage for each cultivar clustered most closely. Samples from ‘unripe’ and ‘turning’ maturity stages from all four cultivars clustered together as did data from the ‘mature’ samples, which formed a separate cluster. Despite producing significantly fewer anthocyanin compounds and in significantly lower quantities than the red-fruited raspberry cultivars, global gene expression data from the ‘mature’ samples of ‘Varnes’ clustered with data from the ‘mature’ samples of the three red-fruited cultivars, indicating a similar global gene expression profile in all four cultivars.

### Differential expression of the major anthocyanin pathway genes

**Fig 2:**
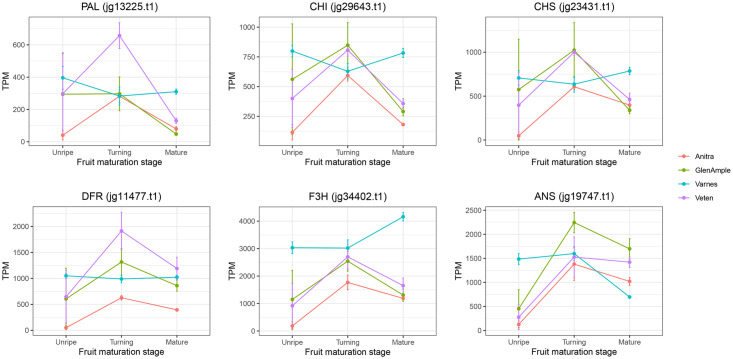
Gene expression profiles (expressed as transcripts per million mapped reads; TPM) of the anthocyanin pathway genes *Phenylalanine lyase* (*Pal*), *Chalcone synthase* (*Chs*), *Chalcone isomerase* (*Chi*), *Flavanone-*β3-hydroxylase (*F3h*), *Dihydroflavanol-4-reductase* (*Dfr*), and *Anthocyanidin synthase* (*Ans*) in four raspberry cultivars, ‘Anitra’, ‘Glen Ample’, ‘Varnes’, and ‘Veten’, at three stages of fruit maturity; ‘unripe’, ‘turning’, and ‘mature’.

The gene expression profiles of the major anthocyanin genes in the four raspberry cultivars ‘Anitra’, ‘Glen Ample’, ‘Varnes’ and ‘Veten’ studied in this investigation are shown in Fig 2. In the three red-fruited cultivars, anthocyanin gene expression was low in the ‘unripe’ samples, significantly increasing and peaking in the ‘turning’ samples before decreasing in the ‘mature’ fruit samples. Gene expression levels in ‘Veten’ were highest in all genes except *Ans*, with ‘Glen Ample’ displaying the highest expression levels for that gene. ‘Anitra’ displayed the lowest gene expression levels for all anthocyanin biosynthesis genes at the ‘unripe’ and ‘turning’ stages, and for all genes at the ‘mature’ phase except *Pal* and *Chs* where ‘Glen Ample’ showed the lowest gene expression. Gene expression for the anthocyanin biosynthesis genes in the ‘Varnes’ samples did not follow the pattern observed in the other three cultivars. Expression of the anthocyanin genes was generally higher in ‘Varnes’ than the red-fruited cultivars and expression levels at each stage were more consistent between stages, with no clear pattern of up- and down-regulation observed as in the red-fruited samples.

**Fig 3:**
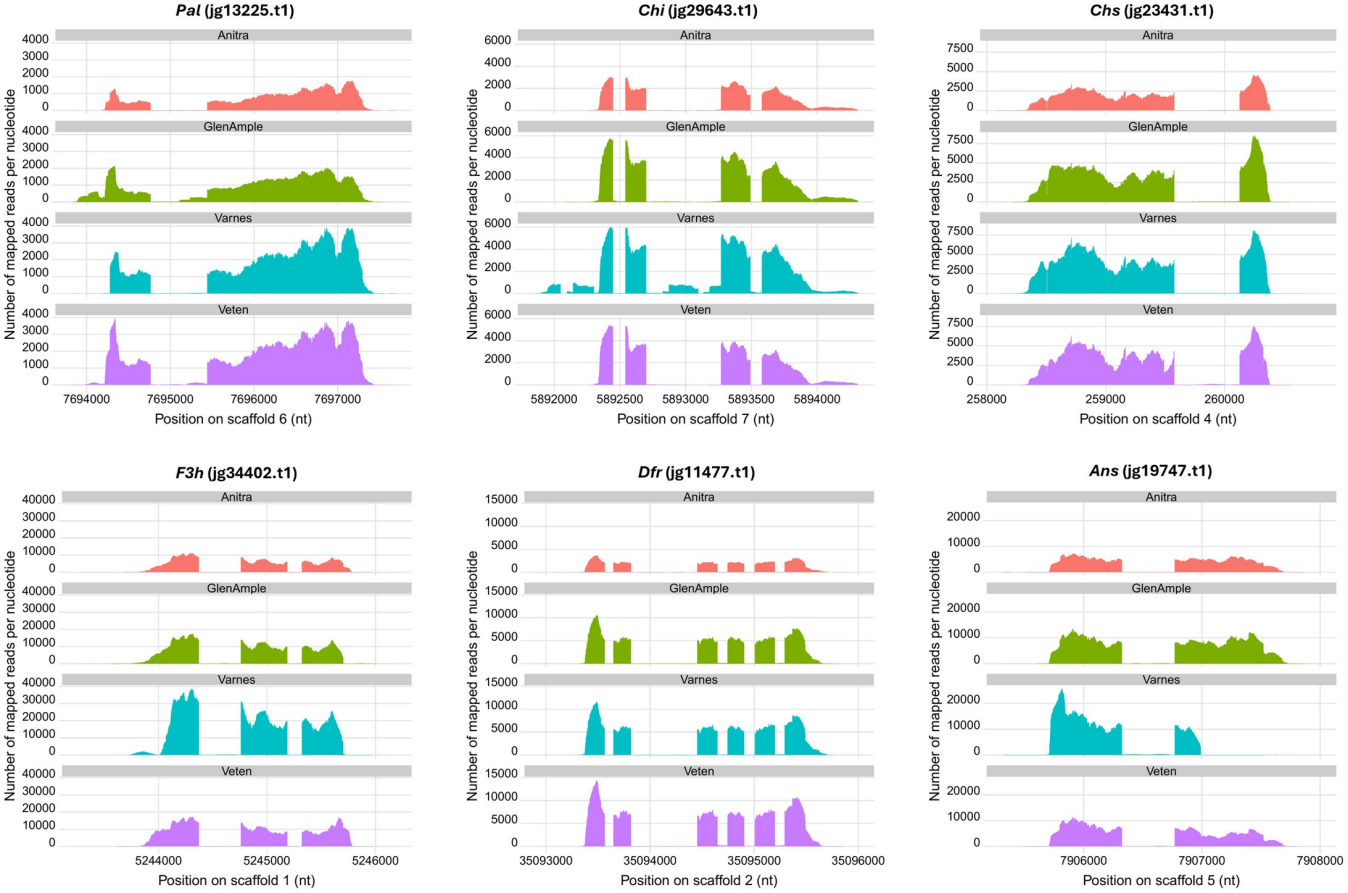
Coverage plots showing the total number of RNASeq reads mapped to the gene predictions from the ‘Malling Jewel’ genome for the anthocyanin genes *Phenylalanine lyase* (*Pal*), *Chalcone synthase* (*Chs*), *Chalcone isomerase* (*Chi*), *Flavanone-*β3-hydroxylase (*F3h*), *Dihydroflavanol-4-reductase* (*Dfr*), and *Anthocyanidin synthase* (*Ans*) in four raspberry cultivars, ‘Anitra’, ‘Glen Ample’, ‘Varnes’, and ‘Veten’. The plot of the *Ans* gene clearly highlights the truncation in the length of the transcribed mRNA in ‘Varnes’.

Scrutiny of the plots of raw reads from all developmental stages mapped to the anthocyanin biosynthesis genes for each cultivar identified similar patterns of read mapping for *Pal*, *Chs*, *Chi*, *F3h* and *Dfr* in all four raspberry cultivars investigated (Fig 3). Analysis of the plots of raw reads mapped against the *Ans* gene showed reads mapping to the entire gene in the three red-fruited cultivars. In ‘Varnes’ however, reads mapped to the first exon and part way through the second exon, but no reads were mapped after nucleotide 7,906,991.

### Assembly and annotation of the ‘Varnes’ genome sequence

A total of 90 Gb of ONT data were returned following sequencing of ‘Varnes’ genomic DNA. The mean length of reads above 10 kb was 24 kb (70.98 Gb, 260× coverage). The longest read was 228,703 bp. Following adapter trimming and filtering for low-quality sequence data, 11.5 Gb of 150 bp paired-end Illumina sequencing data was produced for ‘Varnes’ representing 40× genome coverage.

Following long-read assembly, purging of heterozygous sequence and organelles and polishing, the ‘Varnes’ genome sequence was 276.9 Mb in length and comprised 13 contigs, that all corresponded to sections of the seven chromosomes of the ‘Malling Jewel’ reference sequence (S3 Fig). The *de novo* assembly N_50_ was 37 Mb, with an L_50_ of four contigs and a longest contig sequence of 45.6 Mb. BUSCO analysis returned a 97.9% complete single copy orthologs (S1 Table). A total of 35,773 protein coding genes were identified following annotation and were used for further gene expression analysis using the ‘Varnes’ RNASeq dataset. The raw sequencing data and genome assemblies from the ‘Varnes’ genome were deposited in the NCBI sequence database under the Bioproject ID PRJNA1122082. The ‘Varnes’ assembly was also deposited on the Genome Database for Rosaceae [65].

### Differential expression and structural analysis of the anthocyanidin synthase gene in ‘Varnes’

**Fig 4:**
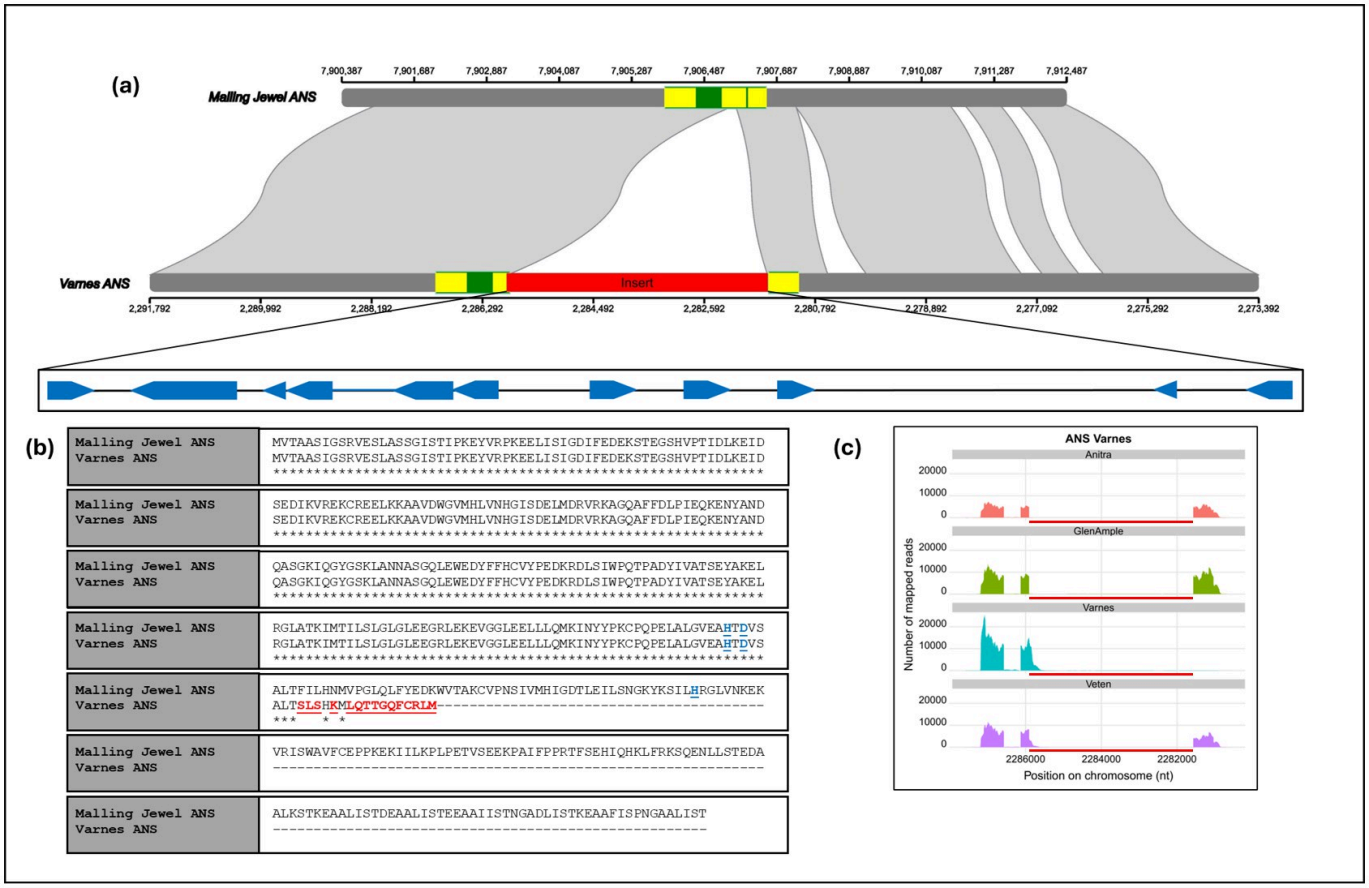
Schematic of the ANS gene region in raspberry cultivars ‘Malling Jewel’ and ‘Varnes’ showing (a) the structure of the gene in the ‘Malling Jewel’ reference, the position of the insert sequence in the ‘Varnes’ sequence and the positions and relative sizes of repeat sequences in the insert; (b) the amino acid sequence of the predicted wild-type protein in ‘Malling Jewel’ and the mutant sequence in ‘Varnes’ (key catalytic residues highlighted in blue, mutated residues highlighted in red); and (c) The abundance of RNASeq reads mapping to the four cultivars (‘Anitra’, ‘Glen Ample’, ‘Varnes’ and ‘Veten’) using the gene predictions from the ‘Varnes’ genome sequence (insertion indicated by red bar).

Structural variant analysis was performed using the ‘Varnes’ sequencing reads against the ‘Malling Jewel’ genome. A total of 42,269 variants were identified in the ‘Varnes’ long-read data, one of which, 4,337 bp in length was located in the *Ans* gene (Fig 4a). Following genome assembly, analysis of the *Ans* gene locus in the ‘Varnes’ genome confirmed the insertion in the *Ans* gene which interrupted the second exon of the gene 512 bp upstream of the ANS-canonical stop codon, introducing a premature stop codon (Fig 4b). The insertion was flanked by a putative three-base target site duplication (CCT) not observed in the wild-type, along with the characteristic CACTA \ TAGTG inverted repeat sequence motif of the En\Spn CACTA class II transposon superfamily, strongly indicating that it is a CACTA-like TE. Despite containing 11 dispersed repeats between 29 nucleotides and 387 nucleotides in length, the insertion did not clearly contain the 10–28 bp terminal inverted repeats (TIRs) normally present in CACTA transposons [66]. This insertion contains typical short sub-terminal repeats (sub-TRs) only in the 5’ region and lacks open reading frames (ORFs) encoding transposase (TnpD) and the regulatory protein TnpA, elements required for autonomous transposition [23]. It seems likely therefore that the element is no longer active within the ‘Varnes’ genome. The element identified was named *RiCACTA1*. Annotation of CACTA elements in the ‘Varnes’ genome sequence with EDTA revealed 455 elements. Phylogenetic analysis of the TIRs of these 455 elements revealed a high degree of homology between *RiCACTA1* and the other elements in the genome (S4 Fig) with *RiCACTA1* clustering as part of a clade containing 15 other CACTA elements.

BLASTn homology using the *RiCACTA1* sequence as a query against ‘Malling Jewel’ reference genome [30] identified five long high homology hits on chromosomes 4, 5, and 6 and two on chromosome 2 that spanned the entire region between the left and right TIRs. All sequences carried TIRs identical to *RiCACTA1*. The elements on chromosome 2 spanned nucleotides 15,794,907–15,798,453, and nucleotides 16,752,860–16,756,069 of the ‘Malling Jewel’ assembly, and were 3,556 bp, and 3,211 bp in length sharing 98.1% and 98.5% identify with *RiCACTA1* respectively. The element on chromosome 4 spanned nucleotides 11,814,389–11,817,932, was 3,551 bp in length and had 98.5% identity with *RiCACTA1*, whilst the element on chromosome 5 spanned nucleotides 12,070,246–12,066,694, was 3,555 bp in length and had 98.5% identity with *RiCACTA1*. Finally, the element on chromosome 6 (nucleotides 26,994,564–26,997,773) spanned 3,211 bp and had an identity of 98.7%, Moreover, there were six partial sequences over 1,000 bp in length that had greater than 87% homology with *RiCACTA1* which were distributed on chromosomes 4, 6 and 7. Corresponding full length sequences with high homology to *RiCACTA1* were found on chromosomes 2, 5 and 7 in the Varnes genome sequence (in addition to the *Ans* gene). In addition, a further seven regions over 1,000 bp in length with a sequence similarity greater than 89% to *RiCACTA1* were found in the ‘Varnes’ genome sequence on chromosomes 1, 5, 6 and 7. From ’Varnes’ and ’Malling Jewel’, all these copies show either crippling deletions and/or extensive nucleotide modifications, supporting the fact that, similarly to *RiCACTA1*, they are no longer able to transpose.

Comparison of the position of the CACTA insertion in ‘Varnes’ with that of the mutation in the yellow-fruited cultivar ‘Anne’ [27] revealed that the two mutations were in the same place in the *Ans* gene. The GGCCT insertion in ‘Anne’ is the typical motif of a transposon insertion and contained the CCT target-site duplication (TSD) observed in ‘Varnes’. Read mapping of the RNASeq data from the four cultivars investigated to the *Ans* gene region in the ‘Varnes’ genome sequence revealed that the first exon and the second exon up to the stop codon were transcribed, but the remaining CDS (Fig 4c) were not. As expected, RNASeq data for ‘Anitra’ and ‘Glen Ample’ mapped exclusively to the sequence of the wild-type *Ans* gene with no reads mapping between nucleotides 2,285,913 and 2,281,573 in the ‘Varnes’ genome sequence, however, RNASeq reads from ‘Veten’ mapped to the full length of the predicted mutant protein in the ‘Varnes’ genome sequence (Fig 4c), indicating that ‘Veten’ is heterozygous for the transposon insertion, whilst ‘Varnes’ is homozygous for the mutation. Expression analysis of the truncated *Ans* gene demonstrated that, as with the other anthocyanin genes in ‘Varnes’, it was not differentially expressed between stages of maturity as it was in the three red fruited cultivars investigated (Fig 2).

The predicted ANS protein sequence from ‘Malling Jewel’ is 414 amino acids in length and contains the highly conserved Fe(2+) 2-oxoglutarate dioxygenase domain of 99 amino acids from position 212 to 311, in which the three characteristic Fe^++^ cation binding sites H-D-H at positions 236, 238 and 292 (indicated in blue characters on Fig 4b) associated with the catalytic properties of that domain are present. The insertion in the ‘Varnes’ *Ans* gene created a truncated predicted protein of 261 amino acids in length with a premature stop codon 50 amino acids before the end of the Fe(2+) 2-oxoglutarate dioxygenase domain. Consequently, the truncated domain in the ‘Varnes’ *Ans* gene does not contain the final histidine residue of the H-D-H motif, and lacks the C-terminal amino acids, including the substrate and co-substrate binding sites of the predicted wild-type protein.

### Confirmation of Ans alleles in ‘Anitra’, ‘Glen Ample’, ‘Varnes’ and ‘Veten’

**Fig 5:**
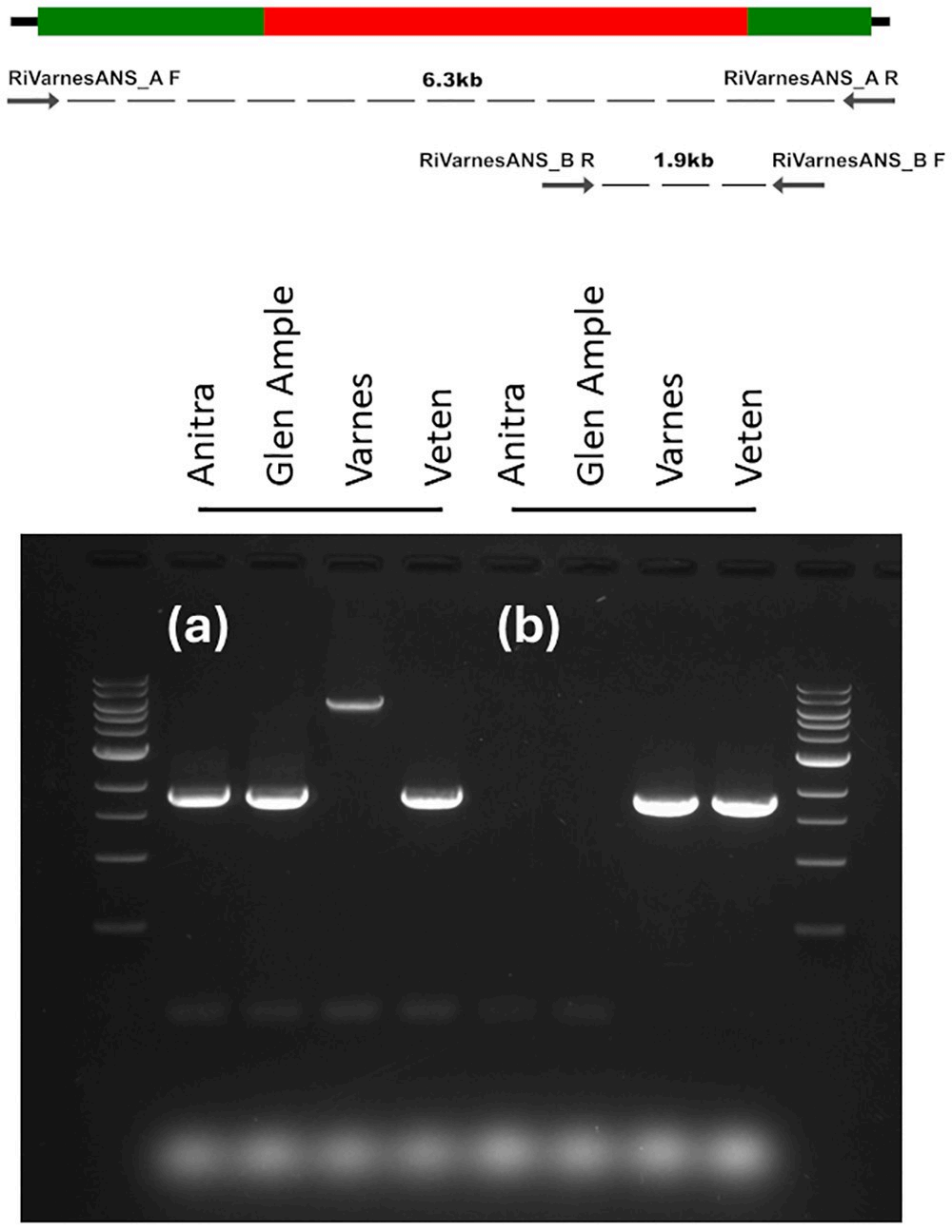
Diagram showing locations of primers designed to amplify the full-length ANS gene (6.3kb) and the insertion-specific amplicon (1.9kb), and agarose gel showing (a) the PCR amplification of a full-length ANS gene from the cultivars ‘Anitra’, ‘Glen Ample’, ‘Varnes’ and ‘Veten’, showing the presence of the wild-type allele (1,837 bp) in ‘Anitra’, ‘Glen Ample’, and ‘Veten’, and the mutant transposon-containing allele (6,173 bp) in ‘Varnes’; (b) the PCR amplification of an ANS transposon specific amplicon (1,819 bp) in cultivars ‘Varnes’ and ‘Veten’.

PCR primer pairs were designed for the full length *Ans* gene (RiVarnesANS_A F: ATG CTC ATT AAA GCA TAA CAA AGG CCC, R: TTA AAC GGC TCC ATT AAT TAA GCA GCA) and an *Ans* insertion-specific product (RiVarnesANS_B F: CTC AGA ATC TCC AAG GTG TCG R: CCC CAA CCT GAA GAA GCA TG) (Fig 5). PCR amplification of the full-length *Ans* gene confirmed the presence of a functional wild-type allele of the *Ans* gene in ‘Anitra’ and ‘Glen Ample’ and ‘Veten’ and the presence of the mutant allele containing the transposon in ‘Varnes’ (Fig 5a). However, amplification of a transposon specific PCR product in ‘Varnes’ and ‘Veten’ confirmed the homozygosity of ‘Varnes’ and the heterozygous genotype of ‘Veten’ and the absence of the transposon-containing allele in ‘Anitra’ and ‘Glen Ample’ (Fig 5b). The lack of amplification of the transposon-containing allele with primer pair RiVarnesANS_A was presumably due to preferential amplification of the smaller wild-type product in this cultivar. The authenticity of the wild-type and transposon-containing PCR products amplified using RiVarnesANS_A and the transposon-specific product using RiVarnesANS_B were confirmed by direct sequencing (S1 File). Although the homology between the amplified products of the two cultivars was very high some single nucleotide and indel polymorphisms were identified.

## Discussion

The genetics of mutations affecting fruit colour in raspberry were first described almost 100 years ago by Crane and Lawrence [5], who reported three distinct fruit-colour types (red, yellow and apricot (or pale)) segregating in a Mendelian fashion suggesting major gene control. The authors noted that fruit and spine colour were inherited together, and that plants with an absence of anthocyanins in their spines could produce either yellow or apricot fruits. It was postulated that colour was controlled by two factors; *T*, which produced anthocyanins in the fruit and spines, and *P* epistatic to *T*, that intensified the colour. Apricot fruits were reported to be due to low levels of anthocyanins produced by the modifier *P* in the absence of *T*.

Recently, a loss of function mutation in the *Ans* gene [27] has been functionally characterised and shown to confer yellow fruits in homozygous form, identifying the *Ans* gene as a good candidate for gene *T* in raspberry as the cultivar studied, ‘Anne’ was devoid of anthocyanins in fruit and spines. The mutant allele of *Ans* containing a CACTA-like TE that was identified in this investigation confers genotypes with apricot coloured fruits. This allele is the likely cause of a partial loss of function mutation leading to fruit with significantly lower levels of anthocyanins than in red-fruited cultivars, and as such is a good candidate for the factor previously described as gene *P*, meaning the *T* and *P* genes [5] are in fact allelic.

The recent rapid advances in long-read genome sequence technologies in terms of depth of coverage and accuracy, in addition to the significant reduction in the cost of generating genome-scale sequence data, have greatly facilitated the discovery and characterisation of mutations underlying phenotypic diversity at the sequence level. The recent publication of chromosome-scale sequence assemblies for raspberry [29, 30] provided a reference for structural variant calling in this investigation, and the utilisation of the long-read Oxford Nanopore sequencing platform enabled the precise identification of a 4.3 kb transposable genetic element in the coding sequence of the *Ans* gene of the raspberry cultivar ‘Varnes’ that was putatively responsible for the apricot colour of the fruit observed.

In several species, it has been reported that genes encoding enzymes and/or transcriptional regulators of anthocyanin biosynthesis have been inactivated by the insertion of members of the CACTA superfamily of TEs. Examples include the *Ans* gene of *Lactuca sativa* [16], the T-DNA *Ans*-tagged gene of *Arabidopsis* [67], both the *Dfr* and *Ans* genes of *Allium cepa* [15], and an active CACTA-like TE found in *Dfr2* in soybean causing variegated flowers [68]. Additionally, Zabala and Vodkin [69] identified an autonomous CACTA-like TE inserted in the soybean *flavonoid 3’-hydroxylase* (*F3’h*) gene, which resulted in a single mutable chimeric plant displaying both tawny and gray trichomes. Following metabolomic analysis, gene expression and whole genome sequencing in this investigation, the 4.3 kb *RiCACTA1* transposable element, flanked by the characteristic CACTA\TAGTG inverted repeat sequence motif of the *En\Spn* CACTA class II transposon superfamily [23], was discovered as the putative cause of the apricot fruit colour in ‘Varnes’.

The terminal regions of all identified CACTA TEs have a similar sequence organization. In particular, CACTA elements have TIRs ranging from 8 to 64 bp, terminating with characteristic CACTA and TAGTG sequences flanked by TSD motifs, and sub-terminal repeats (sub-TRs), ranging from 10 to 20 bp, which are repeated in a direct and inverted orientation [21]. The low sequence conservation of TIRs and sub-TRs makes the identification of CACTA elements difficult unless a transposase (TPase)-like domain is present in the body region. The *RiCACTA1* element identified in ‘Varnes’ lacked the transposase (TnpD) and regulatory protein (TnpA) elements, indicating that it is a non-autonomous element [21]. Interestingly, it did not contain the complete 10–28 bp terminal inverted repeats normally characteristic of CACTA transposons [66], the sequences displaying a nucleotide mismatch proximal to CACTA sequence, and it only contained one sub-TIR region. Altogether, these factors could all have contributed to the lack of mobility of the *RiCACTA1* element.

Rafique *et al*. [27], investigating anthocyanin production in berries of the raspberry cultivar ‘Anne’, demonstrated that the cultivar exhibited a total loss of anthocyanin production, and identified a small 5 bp insertion at nucleotide 745 of the *Ans* coding region that caused a premature stop codon and the prediction of a truncated protein as a result. The insertion in the *Ans* gene in ‘Anne’ had the motif GGCCT, containing a 3 bp TSD (CCT) typical of CACTA TEs [70]. In this investigation, the same CCT target site duplication was identified in the ‘Varnes’ *RiCACTA1* transposon sequence. The presence of this TSD in ‘Anne’ suggests that the same transposition event identified in ‘Varnes’ may also have led to the mutations observed in ‘Anne’, but that the transposon has been excised in yellow-fruited cultivars and could therefore be responsible for both the yellow-fruited and apricot-fruited forms of raspberry characterised at the sequence level.

The study of gene expression in ‘Anne’ compared to the red raspberry cultivar ‘Tulameen’ by Rafique *et al*. [27] revealed a complete loss of expression of the *Ans* gene in ‘Anne’, presumably due to nonsense-mediated mRNA decay mechanisms [71], resulting in the total loss of anthocyanin production in the cultivar. In contrast however, the truncated *Ans* gene in ‘Varnes’ is transcribed and anthocyanin accumulation was observed in the berries. However, only certain anthocyanins were found at the level of detection possible in this experiment, and those that were present were at least 6–8× lower in concentration than what was observed in the red-fruited cultivars studied. Despite being heterozygous for the transposon-containing allele, the ‘Veten’ cultivar showed the highest relative gene expression levels for *Pal*, *Chi*, *Chs*, *F3h* and *Dfr*, but significantly lower levels of relative expression of the *Ans* gene. It also produced the highest concentrations of individual anthocyanins and total anthocyanin content of the fruit, indicating total anthocyanin concentration is not regulated by expression of the *Ans* gene alone.

The functional ANS protein from *Arabidopsis* has a characteristic structure containing a double-stranded β helix topology that forms a hydrophobic cavity housing the active site of the enzyme at one end. At the C-terminal end of the ANS protein is a long loop leading to an α helix that forms a lid over the active site. The active site of the enzyme contains three iron binding residues that ligate an iron molecule in an almost octahedral geometry by the side chains of His-232, Asp-234 and His-288 [72] and it has been reported that the structure of the active site of the ANS:Fe(II):2OG:DHQ relies on the ligation of the Fe(II) atom by the side chains of these three amino acids [72]. In both ‘Anne’ and ‘Varnes’, a premature stop codon in the amino acid sequence results in a protein lacking the His-288 residue, however, the C-terminus of the predicted protein from the two cultivars differs significantly in sequence. The final 19 amino acids of the ANS protein in ‘Anne’ are completely different to the final 18 amino acids of the predicted ANS protein in ‘Varnes’. Whilst both proteins lack the His-288 residue of the active site previously reported to be essential for catalytic activity [72], in contrast to ‘Anne’, the gene in ‘Varnes’ is transcribed at all stages of fruit development. We hypothesise that the *Ans* allele created through the chimerisation of the first 243 amino acids of the *Ans* gene and the 18 amino acids generated by the insertion of the *RiCACTA* TE has residual catalytic activity and permits the biosynthesis of cyanidin-3-sophoroside and cyanidin-3-glucoside in the fruits of the raspberry cultivar ‘Varnes’. Further functional work will need to be performed to determine the catalytic properties of the ANS mutant enzyme and whether it is capable of catalysing anthocyanin biosynthesis in order to fully explain the presence of anthocyanins in the fruit of ‘Varnes’.

Yellow and apricot forms of raspberry have been known for centuries and many yellow and apricot cultivars have been released from breeding programmes globally, indicating the widespread distribution of genetic factors controlling these forms. Colour mutants have been shown to be controlled by major genes in several raspberry species [5–7, 73, 74] and the genes *T* and *P* have been described to explain the genetic control of yellow and apricot forms [5]. The cultivar ‘Veten’ was bred in 1944 at the experimental breeding station at Njøs, Norway from the cross ‘Preussen’ × ‘Lloyd George’. The cultivar ‘Varnes’ was collected as a seed from Puyllaup, Washington, USA in 1987 and is the result of an open pollination of a breeding selection and is therefore from the cross (‘ORUS1846’ × ‘ORUS576/47’) × ‘o.p.’. It is possible that more than one mutation has resulted in yellow raspberry forms, and indeed mutations in any one of the anthocyanin pathway genes could result in loss of function mutations such as that described in ‘Anne’ [27]. Partial loss of function mutations as is observed in ‘Varnes’ in enzymatic pathways however may arise less frequently than loss of function mutations although the other apricot raspberries that have been described would need to be tested to confirm the nature of the mutations they contain. The presence of the transposon-containing allele in the two unrelated cultivars ‘Varnes’ and ‘Veten’ originating from very different sources suggests that this allele may be widespread in raspberry germplasm. Due to the high sequence similarity of these alleles in ‘Varnes’ and ‘Veten’ (97%), it is likely that these alleles originated from the same insertion event, but the nucleotide sequences have since diverged. The PCR primer pairs developed in this investigation will be useful for determining the presence of the allele putatively controlling apricot fruit colour, and its association with the apricot phenotype in a larger sample of germplasm.

## Supporting information

S1 Raw images Original uncropped and unedited gel image used for Fig 5. (PDF)

S1 Fig Principle components analysis of the anthocyanin content of samples of raspberry fruit from four raspberry cultivars, ‘Anitra’ (An), ‘Glen Ample (GA)’, ‘Varnes’ (Va), and ‘Veten’ (Ve), at three stages of fruit maturity; ‘unripe’ (Un), ‘turning’ (Tu), and ‘mature’ (Ma). (TIF)

S2 Fig WCGNA expression cluster tree and anthocyanin biosynthesis heat map showing the relationship between gene expression and anthocyanin production in the 36 fruit samples studied. (PNG)

S3 Fig Alignment of the ‘Varnes’ assembly against the ‘Malling Jewel’ reference genome. (PNG)

S4 Fig Phylogenetic tree of 455 CACTA TIR elements identified in the ‘Varnes’ genome sequence. (PNG)

S1 Table Assembly statistics for the *de novo* assembly of the *Rubus idaeus* ‘Varnes’ genome sequence. (DOCX)

S1 File MAFFT alignment of ‘Varnes’ (Va) and ‘Veten’ (Ve) ANS PCR products to the ANS gene of the ‘Varnes’ genome sequence (Va_ANS_WGS). PCR1 indicates sequence using primer pair RiVarnesANS_A to amplify the full ANS region in ‘Varnes’, whilst PCR2 indicated sequence using primer pair RiVarnesANS_B to amplify the CACTA-specific allele in both ‘Varnes’ and ‘Veten’.
(DOCX)
