# Supplementary figures and images for: A CACTA-like transposon in the Anthocyanidin synthase 1 (Ans-1) gene is responsible for apricot fruit colour in the raspberry (Rubus idaeus) cultivar ‘Varnes’
Source: PLoS One. 2025 Feb 3;20(2):e0318692. doi: 10.1371/journal.pone.0318692 (PMC11790086; doi:10.1371/journal.pone.0318692)

Raw gel image used for Fig5

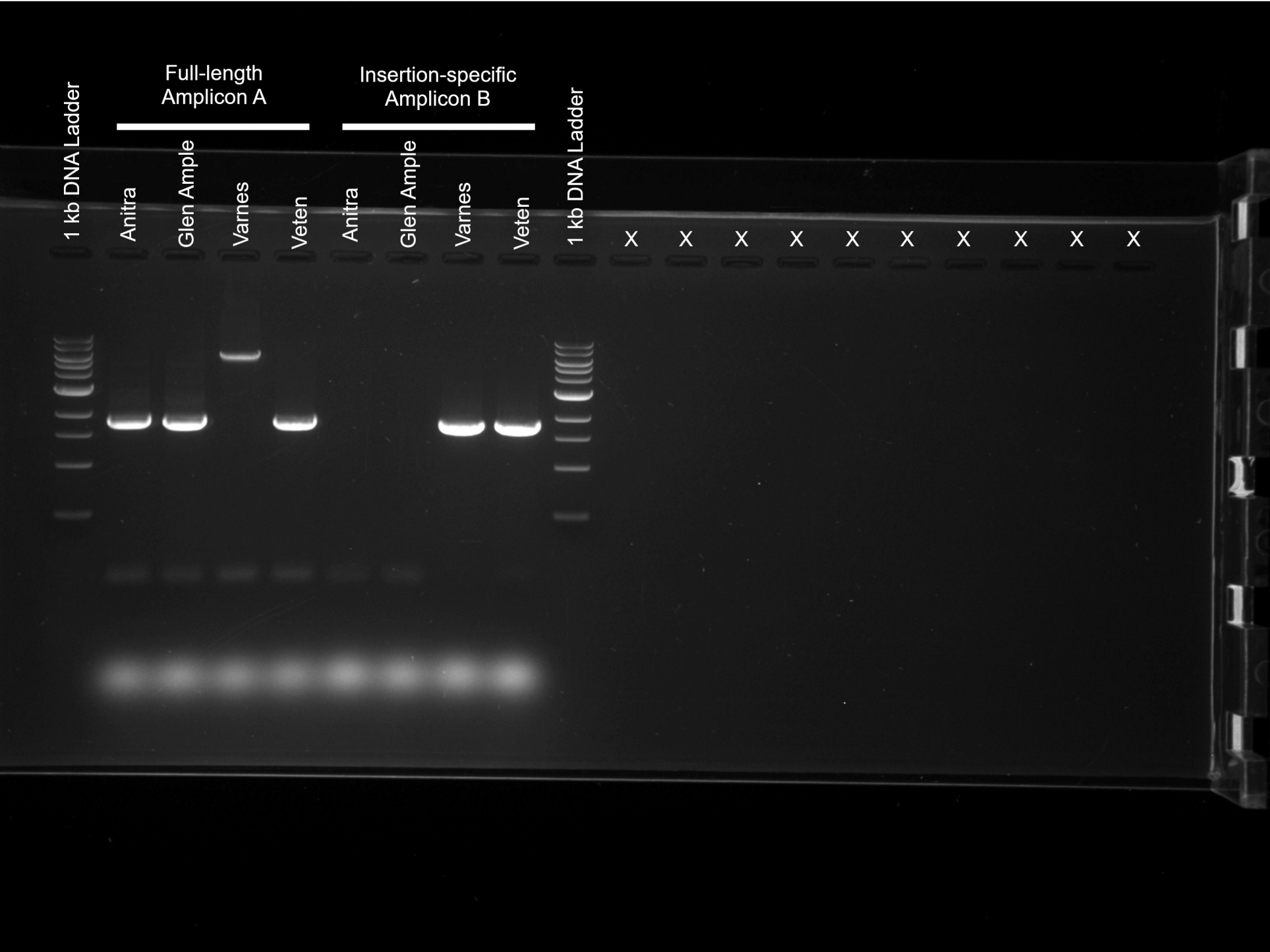

Supplement: S1 Raw images — (PDF) [file pone.0318692.s001.pdf]

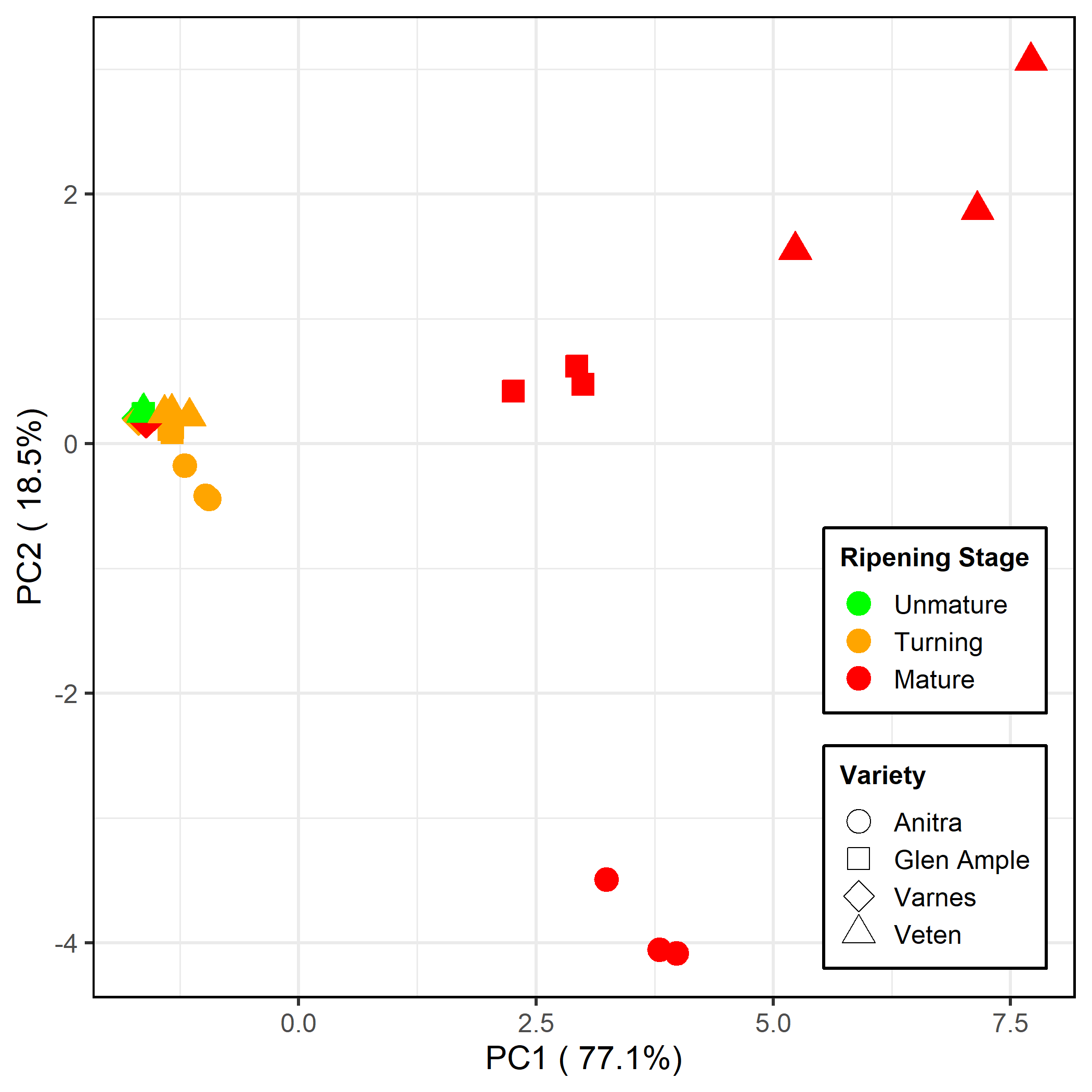

Supplement: S1 Fig — (TIF) [file pone.0318692.s002.tif]

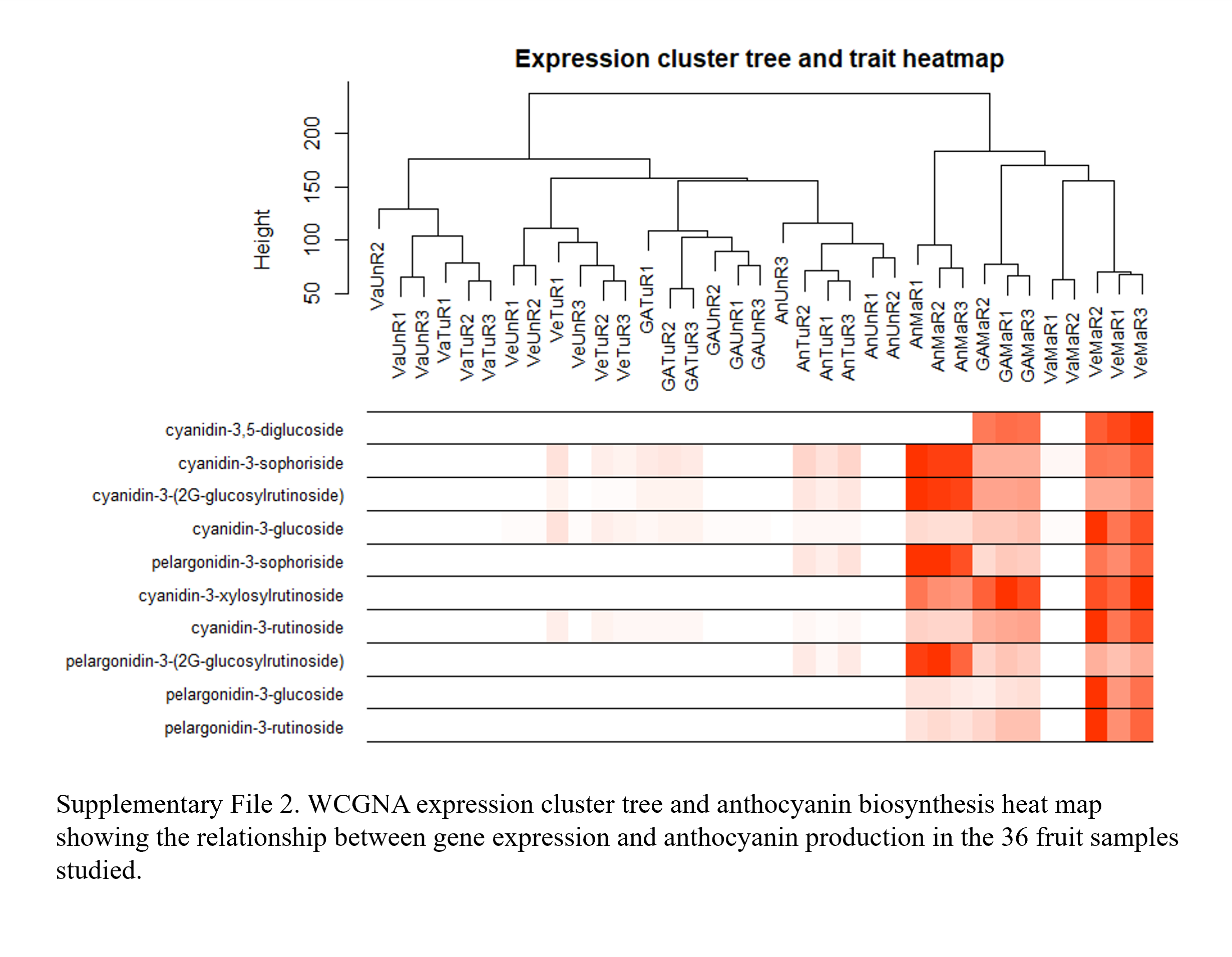

Supplement: S2 Fig — (PNG) [file pone.0318692.s003.png]

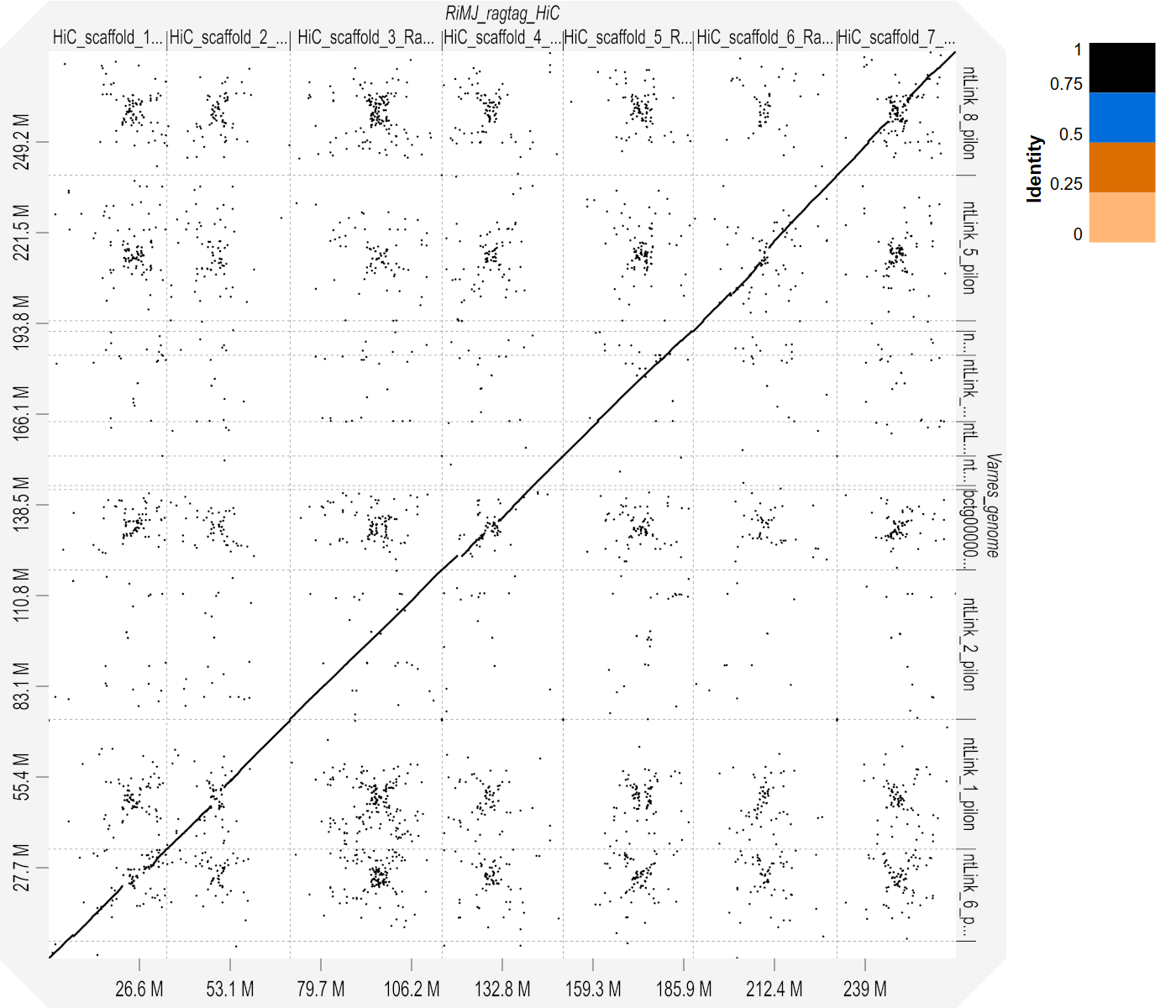

Supplement: S3 Fig — (PNG) [file pone.0318692.s004.png]

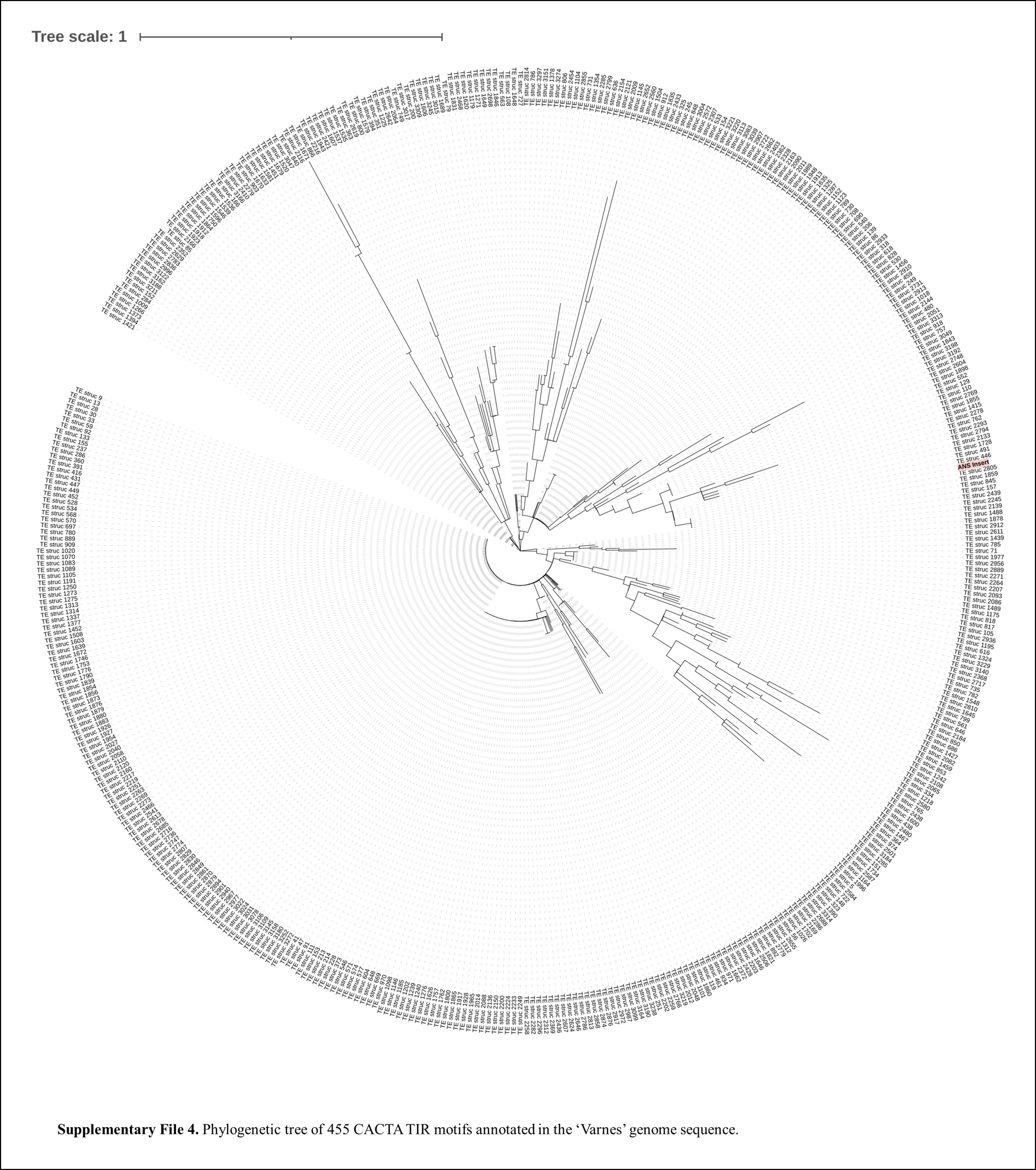

Supplement: S4 Fig — (PNG) [file pone.0318692.s005.png]
