# Supplementary material for: A CACTA-like transposon in the Anthocyanidin synthase 1 (Ans-1) gene is responsible for apricot fruit colour in the raspberry (Rubus idaeus) cultivar ‘Varnes’
Source: PLoS One. 2025 Feb 3;20(2):e0318692. doi: 10.1371/journal.pone.0318692 (PMC11790086; doi:10.1371/journal.pone.0318692)
Supplement: S1 Table — (DOCX) [file pone.0318692.s006.docx]

**Supplementary File 3.** Assembly statistics for the *de novo* assemblies of the *Rubus idaeus* ‘Varnes’ genome sequence.

|  | ‘Varnes’ |
| --- | --- |
| Total sequence length (bp) | 276,910,634 |
| Number of contigs | 13 |
| Longest contig (bp) | 45,624,545 |
| Shortest contig (bp) | 1,247,282 |
| GC content (%) | 37.98 |
| Contig N_50_ (bp) | 37,865,532 |
| Contig L_50_ | 4 |
| Gap (%) | 0.04 |
| Complete BUSCOs (%) | 97.9 |
| Single copy BUSCOs (%) | 95 |
| Duplicated BUSCOs (%) | 2.9 |
| Fragmented BUSCOs (%) | 0.3 |
| Missing BUSCOs (%) | 1.8 |
