# Supplementary material for: A CACTA-like transposon in the Anthocyanidin synthase 1 (Ans-1) gene is responsible for apricot fruit colour in the raspberry (Rubus idaeus) cultivar ‘Varnes’
Source: PLoS One. 2025 Feb 3;20(2):e0318692. doi: 10.1371/journal.pone.0318692 (PMC11790086; doi:10.1371/journal.pone.0318692)
Supplement: S1 File — PCR1 indicates sequence using primer pair RiVarnesANS_A to amplify the full ANS region in ‘Varnes’, whilst PCR2 indicated sequence using primer pair RiVarnesANS_B to amplify the CACTA-specific allele in both ‘Varnes’ and ‘Veten’. (DOCX) [file pone.0318692.s007.docx]

**Supplementary File 6**. MAFFT alignment of ‘Varnes’ (Va) and ‘Veten’ (Ve) ANS PCR products to the ANS gene of the ‘Varnes’ genome sequence (Va_ANS_WGS). PCR1 indicates sequence using primer pair RiVarnesANS_A to amplify the full ANS region in ‘Varnes’, whilst PCR2 indicated sequence using primer pair RiVarnesANS_B to amplify the CACTA-specific allele in both ‘Varnes’ and ‘Veten’. CACTA insertion highlighted in yellow.

CLUSTAL format alignment by MAFFT (v7.511)

VaANS_WGS ------------------------------------------------------------

VaANS_PCR1 atgctcattaaagcataacaaaggccctaggtgcatgtatagcttttgtaaacagcaatt

VaANS_PCR2 ------------------------------------------------------------

VeANS_PCR2 ------------------------------------------------------------

VaANS_WGS ----------------------------------------------------------at

VaANS_PCR1 aaaaaagaacggagagagagagagagagatatcgagagagagctagctataagcaaaaat

VaANS_PCR2 ------------------------------------------------------------

VeANS_PCR2 ------------------------------------------------------------

VaANS_WGS ggtgactgctgcatccattggttcaagagttgagagcttggccagcagcgggatctcgac

VaANS_PCR1 ggtgactgctgcatccattggttcaagagttgagagcttggccagcagcgggatctcgac

VaANS_PCR2 ------------------------------------------------------------

VeANS_PCR2 ------------------------------------------------------------

VaANS_WGS gatcccaaaagagtacgtgagacccaaagaggagctcataagcatcggtgacatctttga

VaANS_PCR1 gatcccaaaagagtacgtgagacccaaagaggagctcataagcatcggtgacatctttga

VaANS_PCR2 ------------------------------------------------------------

VeANS_PCR2 ------------------------------------------------------------

VaANS_WGS ggacgagaagagcactgaggggtctcacgtccccaccattgatttgaaggaaatagactc

VaANS_PCR1 ggacgagaagagcactgaggggtctcacgtccccaccattgatttgaaggaaatagactc

VaANS_PCR2 ------------------------------------------------------------

VeANS_PCR2 ------------------------------------------------------------

VaANS_WGS tgaggacattaaggtgagggagaaatgcagggaggagttgaagaaagcggctgtggattg

VaANS_PCR1 tgaggacattaaggtgagggagaaatgcagggaggagttgaagaaagcggctgtggattg

VaANS_PCR2 ------------------------------------------------------------

VeANS_PCR2 ------------------------------------------------------------

VaANS_WGS gggtgtcatgcacctcgtcaaccatggcatctccgacgagctcatggaccgggtcaggaa

VaANS_PCR1 gggtgtcatgcacctcgtcaaccatggcatctccgacgagctcatggaccgggtcaggaa

VaANS_PCR2 ------------------------------------------------------------

VeANS_PCR2 ------------------------------------------------------------

VaANS_WGS ggccggacaggccttctttgatcttcccattgagcagaaggagaactatgccaatgacca

VaANS_PCR1 ggccggacaggccttctttgatcttcccattgagcagaaggagaactatgccaatgacca

VaANS_PCR2 ------------------------------------------------------------

VeANS_PCR2 ------------------------------------------------------------

VaANS_WGS ggcctcgggcaaaattcaaggctacggaagcaagcttgcaaacaatgcttccgggcaact

VaANS_PCR1 ggcctcgggcaaaattcaaggctacggaagcaagcttgcaaacaatgcttccgggcaact

VaANS_PCR2 ------------------------------------------------------------

VeANS_PCR2 ------------------------------------------------------------

VaANS_WGS tgagtgggaggactactttttccactgtgtgtatcccgaggacaagcgtgacttgtccat

VaANS_PCR1 tgagtgggaggactactttttccactgtgtgtatcccgaggacaagcgtgacttgtccat

VaANS_PCR2 ------------------------------------------------------------

VeANS_PCR2 ------------------------------------------------------------

VaANS_WGS ttggcctcaaacacctgccgattacatgtaagtgttacaaatccatcacttttttacttg

VaANS_PCR1 ttggcctcaaacacctgccgattacatgtaagtgttacaaatccatcacttttttacttg

VaANS_PCR2 ------------------------------------------------------------

VeANS_PCR2 ------------------------------------------------------------

VaANS_WGS ctacttatgacaccaaaataccaatacaatgaatcaccaaccaattatgtacgtccgata

VaANS_PCR1 ctacttatgacaccaaaataccaatacaatgaatcaccaaccaattatgtacgtccgata

VaANS_PCR2 ------------------------------------------------------------

VeANS_PCR2 ------------------------------------------------------------

VaANS_WGS aagtttgtgaatctagctattacttaaatcttgtaagtgttacaaatctacatctctttt

VaANS_PCR1 aagtttgtgaatctagctattacttaaatcttgtaagtgttacaaatctacatctctttt

VaANS_PCR2 ------------------------------------------------------------

VeANS_PCR2 ------------------------------------------------------------

VaANS_WGS acttattacttatgcaaatattttaagaaatagaatctcgatcaatacactggaggaatt

VaANS_PCR1 acttattacttatgcaaatattttaagaaatagaatctcgatcaatacactggaggaatt

VaANS_PCR2 ------------------------------------------------------------

VeANS_PCR2 ------------------------------------------------------------

VaANS_WGS aacaacctattatatatatttcatacaaagtatcatggaagtagcggttacttttttggc

VaANS_PCR1 aacaacctattatatatatttcatacaaagtatcatggaagtagcggttacttttttggc

VaANS_PCR2 ------------------------------------------------------------

VeANS_PCR2 ------------------------------------------------------------

VaANS_WGS tcacacacgtacatcacaaaacactaaaatctcaattacgtaccaaaatcgagttttatt

VaANS_PCR1 tcacacacgtacatcacaaaacactaaaatctcaattacgtaccaaaatcgagttttatt

VaANS_PCR2 ------------------------------------------------------------

VeANS_PCR2 ------------------------------------------------------------

VaANS_WGS tcttttatgtggtatgaatccaccgcttttttggtcacaacacatctcagttttggtaga

VaANS_PCR1 tcttttatgtggtatgaatccaccgcttttttggtcacaacacatctcagttttggtaga

VaANS_PCR2 ------------------------------------------------------------

VeANS_PCR2 ------------------------------------------------------------

VaANS_WGS atgacttctaggtatatattttgctaattgacatcatattcatttgtgtgtagtgtggca

VaANS_PCR1 atgacttctaggtatatattttgctaattgacatcatattcatttgtgtgtagtgtggca

VaANS_PCR2 ------------------------------------------------------------

VeANS_PCR2 ------------------------------------------------------------

VaANS_WGS acaagtgagtacgctaaggaactg-agggggctagcaa-ccaagataatgaccatactct

VaANS_PCR1 acaagtgagtacgctaaggaactgaagggggctagcaacccagaataatgaccatactct

VaANS_PCR2 ------------------------------------------------------------

VeANS_PCR2 ------------------------------------------------------------

VaANS_WGS cacttggcctgggattagaagaagggaggctggagaaggaggtcggtggactcgaagaac

VaANS_PCR1 cacttggcctgggattagaagaagggaggctggagaaggaggtcggtggactcgaagaac

VaANS_PCR2 ------------------------------------------------------------

VeANS_PCR2 ------------------------------------------------------------

VaANS_WGS tcctcctgcaaatgaaaatcaattactacccaaaatgccctcagccggaacttgcactag

VaANS_PCR1 tcctcctgcaaatgaaaatcaattactacccaaaatgccctcagccggaacttgcactag

VaANS_PCR2 ------------------------------------------------------------

VeANS_PCR2 ------------------------------------------------------------

VaANS_WGS gcgtcgaagcccacaccgatgtatctgcactcacctcactaagccacaaaatgcttcaga

VaANS_PCR1 gcgtcgaagcccacaccgatgtatctgcactcacctcactaagccacaaaatgcttcaga

VaANS_PCR2 ------------------------------------------------------------

VeANS_PCR2 ------------------------------------------------------------

VaANS_WGS cgacggggcagttctgtcgtctgatgaactaatttgctgtcgtctactagcctgatatct

VaANS_PCR1 cgacggggcagttctgtcgtctgatgaactaatttgctgtcgtctactagcctgatatct

VaANS_PCR2 ------------------------------------------------------------

VeANS_PCR2 ------------------------------------------------------------

VaANS_WGS gttagcaccatcaaacaacagatattatctgtcgtctaagttataatcaaacaacagaga

VaANS_PCR1 gttagcaccatcaaacaacagatattatctgtcgtctaagttataatcaaacaacagaga

VaANS_PCR2 ------------------------------------------------------------

VeANS_PCR2 ------------------------------------------------------------

VaANS_WGS ttattcattgttgtatatgtaataaaataacagttactgtcgtctaataaaaacgaatta

VaANS_PCR1 ttattcattgttgtatatgtaataaaataacagttactgtcgtctaataaaaacgaatta

VaANS_PCR2 ------------------------------------------------------------

VeANS_PCR2 ------------------------------------------------------------

VaANS_WGS atcttattgtatgatcatttgtggtacttcaacgacagttataagttgtttgaatcaagg

VaANS_PCR1 atcttattgtatgatcatttgtggtacttcaacgacagttataagttgtttgaatcaagg

VaANS_PCR2 ------------------------------------------------------------

VeANS_PCR2 ------------------------------------------------------------

VaANS_WGS ttgaacaacattgggtttataatatatatggtttgaataattttagaatgacattatata

VaANS_PCR1 ttgaacaacattgggtttataatatatatggtttgaataattttagaatgacattatata

VaANS_PCR2 ------------------------------------------------------------

VeANS_PCR2 ------------------------------------------------------------

VaANS_WGS ttgaatttctgtggtttgtagtgaattacaaggacaaatatatgttgtttgatattcctg

VaANS_PCR1 ttgaatttctgtggtttgtagtgaattacaaggacaaatatatgttgtttgatattcctg

VaANS_PCR2 ------------------------------------------------------------

VeANS_PCR2 ------------------------------------------------------------

VaANS_WGS ctaacttcatcgaaactgttgttatcaattgaatcccactacagattttggtttgatttt

VaANS_PCR1 ctaacttcatcgaaactgttgttatcaattgaatcccactacagattttggtttgatttt

VaANS_PCR2 ------------------------------------------------------------

VeANS_PCR2 ------------------------------------------------------------

VaANS_WGS gtaaattgttagtctaacaaacaacaaagtttgggtttggtgtagtttcttatttccata

VaANS_PCR1 gtaaattgttagtctaacaaacaacaaagtttgggtttggtgtagtttcttatttccata

VaANS_PCR2 ------------------------------------------------------------

VeANS_PCR2 ------------------------------------------------------------

VaANS_WGS gacattattcgtttgttatatcatggtcaaacagacatttgagtttgattatgtagtttc

VaANS_PCR1 gacattattcgtttgttatatcatggtcaaacagacatttgagtttgattatgtagtttc

VaANS_PCR2 ------------------------------------------------------------

VeANS_PCR2 ------------------------------------------------------------

VaANS_WGS ttatttcaatggacatcaatcttttgttatttcatggtctaacagacaacagagtttgag

VaANS_PCR1 ttatttcaatggacatcaatcttttgttatttcatggtctaacagacaacagagtttgag

VaANS_PCR2 ------------------------------------------------------------

VeANS_PCR2 ------------------------------------------------------------

VaANS_WGS tttgatatagtttcttattttaatagacatcattcatttgttattacatggtccaacaga

VaANS_PCR1 tttgatatagtttcttattttaatagacatcattcatttgttattacatggtccaacaga

VaANS_PCR2 ------------------------------------------------------------

VeANS_PCR2 ------------------------------------------------------------

VaANS_WGS caacagagtttgagtttgattatgtagtttcttatttcaatagacatcaatcttttgtta

VaANS_PCR1 caacagagtttgagtttgattatgtagtttcttatttcaatagacatcaatcttttgtta

VaANS_PCR2 ------------------------------------------------------------

VeANS_PCR2 ------------------------------------------------------------

VaANS_WGS tttcatggtctatcagacaacagagtttgagtttgatgtagtttcttattttaatagaca

VaANS_PCR1 tttcatggtctatcagacaacagagtttgagtttgatgtagtttcttattttaatagaca

VaANS_PCR2 ------------------------------------------------------------

VeANS_PCR2 ------------------------------------------------------------

VaANS_WGS tcattcatttgttattacatggtccaacagacaacagcatttgaatttgattatgtagtt

VaANS_PCR1 tcattcatttgttattacatggtccaacagacaacagcatttgaatttgattatgtagtt

VaANS_PCR2 ------------------------------------------------------------

VeANS_PCR2 ------------------------------------------------------------

VaANS_WGS tcttatttcaatagacatcaatcctttattgtttcataaccatatacacaacttatttac

VaANS_PCR1 tcttatttcaatagacatcaatcctttattgtttcataaccatatacacaacttatttac

VaANS_PCR2 ------------------------------------------------------------

VeANS_PCR2 ------------------------------------------------------------

VaANS_WGS gtatgcccctgtcatttgatatgataatctggaaactagcagctagttacgaaaatcctt

VaANS_PCR1 gtatgcccctgtcatttgatatgataatctggaaactagcagctagttacgaaaatcctt

VaANS_PCR2 ------------------------------------------------------------

VeANS_PCR2 ------------------------------------------------------------

VaANS_WGS atttataataatctgttgatccatattcattaagttcataatccatattgtgtgcacaac

VaANS_PCR1 atttataataatctgttgatccatattcattaagttcataatccatattgtgtgcacaac

VaANS_PCR2 ------------------------------------------------------------

VeANS_PCR2 ------------------------------------------------------------

VaANS_WGS aaaatacattcataataaggtccatctgtgattaattactaaacccatgaacaaaaggat

VaANS_PCR1 aaaatacattcataataaggtccatctgtgattaattactaaacccatgaacaaaaggat

VaANS_PCR2 ------------------------------------------------------------

VeANS_PCR2 ------------------------------------------------------------

VaANS_WGS cgatcaatatatataaggctgcaacacagaatacaacacaatcataccaaaaacaaagac

VaANS_PCR1 cgatcaatatatataaggctgcaacacagaatacaacacaatcataccaaaaacaaagac

VaANS_PCR2 ------------------------------------------------------------

VeANS_PCR2 ------------------------------------------------------------

VaANS_WGS caactgtttgattttccatgggttactcacaaaatgaggtcctaaccaataagatctagt

VaANS_PCR1 caactgtttgattttccatgggttactcacaaaatgaggtcctaaccaataagatctagt

VaANS_PCR2 ------------------------------------------------------------

VeANS_PCR2 ------------------------------------------------------------

VaANS_WGS tatttggaaacttcacaacatgatttgcccactcagcccagacttcatcgatgtcctcct

VaANS_PCR1 tatttggaaacttcacaacatgatttgcccactcagcccagacttcatcgatgtcctcct

VaANS_PCR2 ------------------------------------------------------------

VeANS_PCR2 ------------------------------------------------------------

VaANS_WGS gtgtgtatttcaatgcatttcttctttcccactgcgataataaaaactatgcaagttatg

VaANS_PCR1 gtgtgtatttcaatgcatttcttctttcccactgcgataataaaaactatgcaagttatg

VaANS_PCR2 ------------------------------------------------------------

VeANS_PCR2 ------------------------------------------------------------

VaANS_WGS atgaaatcaaattcattcctaagccctatataattaaactatatatcatgttgcatatat

VaANS_PCR1 atgaaatcaaattcattcctaagccctatataattaaactatatatcatgttgcatatat

VaANS_PCR2 ------------------------------------------------------------

VeANS_PCR2 ------------------------------------------------------------

VaANS_WGS aaacagcacacaattcaaacacagcacaacacacaattagaacacatggctgatagttta

VaANS_PCR1 aaacagcacacaattcaaacacagcacaacacacaattagaacacatggctgatagttta

VaANS_PCR2 ------------------------------------------------------------

VeANS_PCR2 ------------------------------------------------------------

VaANS_WGS aacatgcatttgcatacttaatggataaaagaaacacaattgcctaccaaacatctaaaa

VaANS_PCR1 aacatgcatttgcatacttaatggataaaagaaacacaattgcctaccaaacatctaaaa

VaANS_PCR2 ------------------------------------------------------------

VeANS_PCR2 ------------------------------------------------------------

VaANS_WGS tcagacatgacacattaccttgacaccaaactccaagttcgtgtcctctataatctcctt

VaANS_PCR1 tcagacatgacacattaccttgacaccaaactccaagttcgtgtcctctataatctcctt

VaANS_PCR2 ------------------------------------------------------------

VeANS_PCR2 ------------------------------------------------------------

VaANS_WGS catataacgcattatgaaatacccacagtccttgtcaccattttgggtcggaatgccctg

VaANS_PCR1 catataacgcattatgaaatacccacagtccttgtcaccattttgggtcggaatgccctg

VaANS_PCR2 ------------------------------------------------------------

VeANS_PCR2 ------------------------------------------------------------

VaANS_WGS caaatatatcattaattaagtacctagtgttagaaccaaatctaaaaccttaaattaagg

VaANS_PCR1 caaatatatcattaattaagtacctagtgttagaaccaaatctaaaaccttaaattaagg

VaANS_PCR2 ------------------------------------------------------------

VeANS_PCR2 ------------------------------------------------------------

VaANS_WGS agacgacaaaggataagaacaatgtagattttaatataatcttgctagataagtttggac

VaANS_PCR1 agacgacaaaggataagaacaatgtagattttaatataatcttgctagataagtttggac

VaANS_PCR2 ------------------------------------------------------------

VeANS_PCR2 ------------------------------------------------------------

VaANS_WGS atagtttactactcacacaaacagtaatatgtaagatttcagtaatcagcttttagattt

VaANS_PCR1 atagtttactactcacacaaacagtaatatgtaagatttcagtaatcagcttttagattt

VaANS_PCR2 ------------------------------------------------------------

VeANS_PCR2 ------------------------------------------------------------

VaANS_WGS tttttgatgtgaagacttaacttcgaaatgtcataacttaatctagaaaatttagtttca

VaANS_PCR1 tttttgatgtgaagacttaacttcgaaatgtcataacttaatctagaaaatttagtttca

VaANS_PCR2 ------------------------------------------------------------

VeANS_PCR2 ------------------------------------------------------------

VaANS_WGS ggatattcaaagtctgaaataatctttgagaggtacactacaactttcatgaagactcaa

VaANS_PCR1 ggatattcaaagtctgaaataatctttgagaggtacactacaactttcatgaagactcaa

VaANS_PCR2 ------------------------------------------------------------

VeANS_PCR2 ------------------------------------------------------------

VaANS_WGS acttcaaaaacccaacagaattgtatagtttttaacaaagagttcactaggtcagaaact

VaANS_PCR1 acttcaaaaacccaacagaattgtatagtttttaacaaagagttcactaggtcagaaact

VaANS_PCR2 ------------------------------------------------------------

VeANS_PCR2 ------------------------------------------------------------

VaANS_WGS cagaaaatgagtaactataacggtaactaattaccttcacagttcttcacttcttccact

VaANS_PCR1 cagaaaatgagtaactataacggtaactaattaccttcacagttcttcacttcttccact

VaANS_PCR2 ------------------------------------------------------------

VeANS_PCR2 ------------------------------------------------------------

VaANS_WGS aaagaatcccataacctaaaaagagagcaaattgaaaacaatatatgcaagattaatatg

VaANS_PCR1 aaagaatcccataacctaaaaagagagcaaattgaaaacaatatatgcaagattaatatg

VaANS_PCR2 ------------------------------------------------------------

VeANS_PCR2 ------------------------------------------------------------

VaANS_WGS gaagatgaaatgtaaataatcacagagaacaatctttcaaggaacagaagaggatatata

VaANS_PCR1 gaagatgaaatgtaaataatcacagagaacaatctttcaaggaacagaagaggatatata

VaANS_PCR2 ------------------------------------------------------------

VeANS_PCR2 ------------------------------------------------------------

VaANS_WGS ttgttatcaattataatgatattgaaacttcctgatgtaaagctttaacttcaaaatttc

VaANS_PCR1 ttgttatcaattataatgatattgaaacttcctgatgtaaagctttaacttcaaaatttc

VaANS_PCR2 ------------------------------------------------------------

VeANS_PCR2 ------------------------------------------------------------

VaANS_WGS ataacttaatctagaaatttcatttttaggagtttcaaagtctgaaattattttaaagat

VaANS_PCR1 ataacttaatctagaaatttcatttttaggagtttcaaagtctgaaattattttaaagat

VaANS_PCR2 ------------------------------------------------------------

VeANS_PCR2 ------------------------------------------------------------

VaANS_WGS gtctactacaactttcatgaaggctctaacttcaaatacccatcagaattgtatagttta

VaANS_PCR1 gtctactacaactttcatgaaggctctaacttcaaatacccatcagaattgtatagttta

VaANS_PCR2 ------------------------------------------------------------

VeANS_PCR2 ------------------------------------------------------------

VaANS_WGS taccaaaaagatcactgggtcagaaactcagaaaaactgactgtgtttaccaatttttca

VaANS_PCR1 taccaaaaagatcactgggtcagaaactcagaaaaactgactgtgtttaccaatttttca

VaANS_PCR2 ------------------------------------------------------------

VeANS_PCR2 ------------------------------------------------------------

VaANS_WGS aagtgttcatgtgtttagctaacagaagcatatatataagcacaaattattaacactgca

VaANS_PCR1 aagtgttcatgtgtttagctaacagaagcatatatataagcacaaattattaacactgca

VaANS_PCR2 ------------------------------------------------------------

VeANS_PCR2 ------------------------------------------------------------

VaANS_WGS aacataagctgaagggtgtcatgtgacctattattacaaatttaggatgtctactgtaaa

VaANS_PCR1 aacataagctgaagggtgtcatgtgacctattattacaaatttaggatgtctactgtaaa

VaANS_PCR2 ------------------------------------------------------------

VeANS_PCR2 ------------------------------------------------------------

VaANS_WGS acacacttgctataggggattaaaccctatgcctaagaagcaaatcaaatatatgtaaag

VaANS_PCR1 acacacttgctataggggattaaaccctatgcctaagaagcaaatcaaatatatgtaaag

VaANS_PCR2 ------------------------------------------------------------

VeANS_PCR2 ------------------------------------------------------------

VaANS_WGS acaaactcataagccttcaaaaattcaactatatgcatcaccccaacctgaagaagcatg

VaANS_PCR1 acaaactcataagccttcaaaaattcaactatatgcatcaccccaacctgaagaagcatg

VaANS_PCR2 ------------------------------------------------------------

VeANS_PCR2 ------------------------------------------------------------

VaANS_WGS cagccccgacatcaacacaaacacaagaaaat-gctgcagcttaaacaaaaaatcat-gc

VaANS_PCR1 cagccccgacatcaacacaaacacaagaaaat-gctgcagcttaaacaaaaaatcat-gc

VaANS_PCR2 --------------acacaaacacaagaaaat-gctgcagcttaaacaaaaaatcat-gc

VeANS_PCR2 --------------taacaaacacaagaaaatggctgcagctttaacaaaaaatcatggc

**************** ********** ************* **

VaANS_WGS agcaaactttgaaatactcattaaggtgtctactacaaattctgagaagacaacaacttc

VaANS_PCR1 agcaaactttgaaatactcattaaggtgtctactacaaattctgagaagacaacaacttc

VaANS_PCR2 agcaaactttgaaatactcattaaggtgtctactacaaattctgagaagacaacaacttc

VeANS_PCR2 agcaaactttgaaatactcattaaggtgtctactacaaattctgagaagacaacaacttc

************************************************************

VaANS_WGS aaattatgaacgaaattgtactgtttttaaggaaacaggttgctgggtcataatactgca

VaANS_PCR1 aaattatgaacgaaattgtactgtttttaaggaaacaggttgctgggtcataatactgca

VaANS_PCR2 aaattatgaacgaaattgtactgtttttaaggaaacaggttgctgggtcataatactgca

VeANS_PCR2 aaattatgaacgaaattgtactgtttttaaggaaacaggttgctgggtcataatactgca

************************************************************

VaANS_WGS gttatatgaaaccaattgggtttagaaacacccataattaaaatatgaaaacttaagcat

VaANS_PCR1 gttatatgaaaccaattgggtttagaaacacccataattaaaatatgaaaacttaagcat

VaANS_PCR2 gttatatgaaaccaattgggtttagaaacacccataattaaaatatgaaaacttaagcat

VeANS_PCR2 gttatatgaaaccaattgggtttagaaacacccataattaaaatatgaaaacttaagcat

************************************************************

VaANS_WGS gataaaatctgtacactacaagaataaagagattggcaatagaagtaagagctctcacat

VaANS_PCR1 gataaaatctgtacactacaagaataaagagattggcaatagaagtaagagctctcacat

VaANS_PCR2 gataaaatctgtacactacaagaataaagagattggcaatagaagtaagagctctcacat

VeANS_PCR2 gataaaatctgtacactacaagaataaagagattggcaatagaagtaagagctctcacat

************************************************************

VaANS_WGS aaagagaaaagaacataggaaattgacatctgccaattgtgccaactctaacttcaaatc

VaANS_PCR1 aaagagaaaagaacataggaaattgacatctgccaattgtgccaactctaacttcaaatc

VaANS_PCR2 aaagagaaaagaacataggaaattgacatctgccaattgtgccaactctaacttcaaatc

VeANS_PCR2 aaagagaaaagaacataggaaattgacatctgccaattgtgccaactctaacttcaaatc

************************************************************

VaANS_WGS catctcaagctaaaccaaaaggaattttggtaggacaaaacctctattttcttgtcaaac

VaANS_PCR1 catctcaagctaaaccaaaaggaattttggtaggacaaaacctctattttcttgtcaaac

VaANS_PCR2 catctcaagctaaaccaaaaggaattttggtaggacaaaacctctattttcttgtcaaac

VeANS_PCR2 catctcaagctaaaccaaaaggaattttggtaggacaaaacctctattttcttgtcaaac

************************************************************

VaANS_WGS aaaatgaaaagattagtatcaacggtgataagaacataagaacatagactcactttctct

VaANS_PCR1 aaaatgaaaagattagtatcaacggtgataagaacataagaacatagactcactttctct

VaANS_PCR2 aaaatgaaaagattagtatcaacggtgataagaacataagaacatagactcactttctct

VeANS_PCR2 aaaatgaaaagattagtatcaacggtgataagaacataagaacatagactcactttctct

************************************************************

VaANS_WGS attaatttaagactcacagttcacaacttcacataatcacaattcataagttcacaacac

VaANS_PCR1 attaatttaagactcacagttcacaacttcacataatcacaattcataagttcacaacac

VaANS_PCR2 attaatttaagactcacagttcacaacttcacataatcacaattcataagttcacaacac

VeANS_PCR2 attaatttaagactcacagttcacaacttcacataatcacaattcataagttcacaacac

************************************************************

VaANS_WGS agacacactcatatactgcctagttcaataaatcaatatcacaaattcacaggaccaaac

VaANS_PCR1 agacacactcatatactgcctagttcaataaatcaatatcacaaattcacaggaccaaac

VaANS_PCR2 agacacactcatatactgcctagttcaataaatcaatatcacaaattcacaggaccaaac

VeANS_PCR2 agacacactcatatactgcctagttcaataaatcaatatcacaaattcacaggaccaaac

************************************************************

VaANS_WGS tagcagctcgggttcattaatcaatcaatcaaactagcagcagcaaaatagcagactagc

VaANS_PCR1 tagcagctcgggttcattaatcaatcaatcaaactagcagcagcaaaatagcagactagc

VaANS_PCR2 tagcagctcgggttcattaatcaatcaatcaaactagcagcagcaaaatagcagactagc

VeANS_PCR2 tagcagctcgggttcattaatcaatcaatcaaactagcagcagcaaaatagcagactagc

************************************************************

VaANS_WGS agcagctcgggttcaaaataaatacagatagagccacaaattatgaaaatcaaaatggta

VaANS_PCR1 agcagctcgggttcaaaataaatacagatagagccacaaattatgaaaatcaaaatggta

VaANS_PCR2 agcagctcgggttcaaaataaatacagatagagccacaaattatgaaaatcaaaatggta

VeANS_PCR2 agcagctcgggttcaaaataaatacagatagagccacaaattatgaaaatcaaaatggta

************************************************************

VaANS_WGS ccttgatgaggattcggtcgttgtaggagcttgtccatggtctcggggagaagtgcgaag

VaANS_PCR1 ccttgatgaggattcggtcgttgtaggagcttgtccatggtctcggggagaagtgcgaag

VaANS_PCR2 ccttgatgaggattcggtcgttgtaggagcttgtccatggtctcggggagaagtgcgaag

VeANS_PCR2 ccttgatgaggattcggtcgttgtaggagcttgtccatggtctcggggagaagtgcgaag

************************************************************

VaANS_WGS tgcgaagacattcaccaaaatacaaacaagacctctaaaactagaaccaagaaagaagta

VaANS_PCR1 tgcgaagacattcaccaaaatacaaacaagacctctaaaactagaaccaagaaagaagta

VaANS_PCR2 tgcgaagacattcaccaaaatacaaacaagacctctaaaactagaaccaagaaagaagta

VeANS_PCR2 tgcgaagacattcaccaaaatacaaacaagacctcttaaactagacccccgaaagaagta

************************************ ******** ** **********

VaANS_WGS aaaattcaatttgcaaagaaaaaacaaatcttgaaataaagggagaacctttccaccgag

VaANS_PCR1 aaaattcaatttgcaaagaaaaaacaaatcttgaaataaagggagaacctttccaccgag

VaANS_PCR2 aaaattcaatttgcaaagaaaaaacaaatcttgaaataaagggagaacctttccaccgag

VeANS_PCR2 aaaattcaatttgcacagagaaaacacatcttgagataaagggagaacctttcccccgag

*************** ***.****** *******.******************* *****

VaANS_WGS atagatcttctccacatcgagggagatggcattgattgattcagtcatggctaagctctc

VaANS_PCR1 atagatcttctccacatcgagggagatggcattgattgattcagtcatggctaagctctc

VaANS_PCR2 atagatcttctccacatcgagggagatggcattgattgattcagtcatggctaagctctc

VeANS_PCR2 atagatcttctccacatcgagggagatggcattgattgattcagtcatgggtaagctctc

************************************************** *********

VaANS_WGS ctagcgcgcagcacacagagggcttcgctatcttcttcctctctgagcaaacctcactgc

VaANS_PCR1 ctagcgcgcagcacacagagggcttcgctatcttcttcctctctgagcaaacctcactgc

VaANS_PCR2 ctagcgcgcagcacacagagggcttcgctatcttcttcctctctgagcaaacctcactgc

VeANS_PCR2 ttagcgcgcagcacacagaggggtttgctatcttcttcttctctgagcaaacctcactgt

.********************* **.************.********************.

VaANS_WGS tcctacatccctcggcacctcaaaccatgagcttctccctcatctctcagaaaggagcta

VaANS_PCR1 tcctacatccctcggcacctcaaaccatgagcttctccctcatctctcagaaaggagcta

VaANS_PCR2 tcctacatccctcggcacctcaaaccatgagcttctccctcatctctcagaaaggagcta

VeANS_PCR2 tcttacatccctcggcacctcaaaccatgaggttttccctcatttttcagaaaggagcta

**.**************************** **.********.*.**************

VaANS_WGS ctgggttcatcgatatgggttgtgattgacagattgaggggttctag---tttttttttt

VaANS_PCR1 ctgggttcatcgatatgggttgtgattgacagattgaggggttctag---tttttttttt

VaANS_PCR2 ctgggttcatcgatatgggttgtgattgacagattgaggggttctag---tttttttttt

VeANS_PCR2 ttgggtttttttatatgggttgtgtttgacagattgaggggttctagttttttttttttt

.******. *. ************ ********************** **********

VaANS_WGS ttggtggtttttggctgaactattgaattgatactgaatagaaatgatgttggctaagaa

VaANS_PCR1 ttggtggtttttggctgaactattgaattgatactgaatagaaatgatgttggctaagaa

VaANS_PCR2 ttggtggtttttggctgaactattgaattgatactgaatagaaatgatgttggctaagaa

VeANS_PCR2 ttgggggtttttgggtgaaatattgaattgatacagaatagaaaaaatgttgggtaaaaa

**** ********* **** ************** ********* .******* ***.**

VaANS_WGS atccttgggtttgatacgaatgttagctaatcgaaatccaaacgaagaagtgaagatttt

VaANS_PCR1 atccttgggtttgatacgagtgttagctaatcgaaatccaaacgaagaagtgaagatttt

VaANS_PCR2 atccttgggtttgatacgagtgttagctaatcgaaatccaaacgaagaagtgaagatttt

VeANS_PCR2 atccttgggtttgatacgaatgttagataatagaaatccaaacgaagaagagaagatttt

*******************.****** **** ****************** *********

VaANS_WGS ttttctttgttcttcgatgagtggcgagagagagagagagagagagagagagagttggaa

VaANS_PCR1 ttttttttgtttttcgatgagtggcgagagagagagagagagagagagagagagttggaa

VaANS_PCR2 ttttttttgtttttcgatgagtggcgagagagagagagagagagagagagagagttggaa

VeANS_PCR2 tttttttttttttttgttgagtggggagagagagagagagagagagagagagagttggaa

****.*** **.**.* ******* ***********************************

VaANS_WGS gggaggtcatgtgtgtaggctgctcgagagggaaaccaaatattagtgagggtttgttga

VaANS_PCR1 gggaggtcatgtgtgtaggctgctcgagagggaaaccaaatattagtgagggtttgttga

VaANS_PCR2 gggaggtcatgtgtgtaggctgctcgagagggaaaccaaatattagtgagggtttgttga

VeANS_PCR2 gggaggtcatgtgtgtaggctgctcgagagggaaaccaaatattagtgagggtttgttga

************************************************************

VaANS_WGS gagggaaacctcaaaaaatttgaagtgtgggaaccctaacaaatatgcttaaaatgacgt

VaANS_PCR1 gagggaaacctcaaaaaatttgaagtgtgggaaccctaacaaatatgcttaaaatgacgt

VaANS_PCR2 gagggaaacctcaaaaaatttgaagtgtgggaaccctaacaaatatgcttaaaatgacgt

VeANS_PCR2 gagggaaacctcaaaaaatttgaagtgtgggaaccctaacaaatatgcttaaaatgacgt

************************************************************

VaANS_WGS aacccccaaaaaaaaaactggacttttttgggtgatgagatttttcagacaacatatagt

VaANS_PCR1 aacccccaaaaaaaaaactggacttttttgggtgatgagatttttcagacaacatatagt

VaANS_PCR2 aacccccaaaaaaaaaactggacttttttgggtgatgagatttttcagacaacatatagt

VeANS_PCR2 aacccccaaaaaaaaaactggacttttttgggtgatgagatttttcagacaacatatagt

************************************************************

VaANS_WGS catagaactgtcgtctaaacacacccaactgatgtcccgcttctataaaaaaatttaggt

VaANS_PCR1 catagaactgtcgtctaaacacacccaactgatgtcccgcttctataaaaaaatttaggt

VaANS_PCR2 catagaactgtcgtctaaacacacccaactgatgtcccgcttctataaaaaaatttaggt

VeANS_PCR2 catagaactgtcgtctaaacacacccaactgatgtcccgcttctataaaaaaatttaggt

************************************************************

VaANS_WGS atgacgtataccactatcagacgacatacttaatttatatgtcgtttgaaaatttcagac

VaANS_PCR1 atgacgtataccactatcagacgacatacttaatttatatgtcgtttgaaaatttcagac

VaANS_PCR2 atgacgtataccactatcagacgacatacttaatttatatgtcgtttgaaaatttcagac

VeANS_PCR2 atgacgtataccactatcagacgacatacttaatttatatgtcgtttgaaaatttcagac

************************************************************

VaANS_WGS aacagaaaatatgaattctgtcgttaaacatgtgtcgtctgataacattattggcatagt

VaANS_PCR1 aacagaaaatatgaattctgtcgttaaacatgtgtcgtctgataacattattggcatagt

VaANS_PCR2 aacagaaaatatgaattctgtcgttaaacatgtgtcgtctgataacattattggcatagt

VeANS_PCR2 aacagaaaatatgaattctgtcgttaaacatgtgtcgtctgataacattattggcatagt

************************************************************

VaANS_WGS gccttcatcctccacaacatggttcccggcctgcagctcttctacgaagacaaatgggtg

VaANS_PCR1 gccttcatcctccacaacatggttcccggcctgcagctcttctacgaagacaaatgggtg

VaANS_PCR2 gccttcatcctccacaacatggttcccggcctgcagctcttctacgaagacaaatgggtg

VeANS_PCR2 gccttcatcctccacaacatggttcccggcctgcagctcttctacgaagacaaatgggtg

************************************************************

VaANS_WGS acggcgaaatgcgtcc-ccaactccatcgtcatgcacatcggcgacaccttggagattct

VaANS_PCR1 acggcgaaatgcgtcc-ccaactccatcgtcatgcacatcggcgacaccttggagattct

VaANS_PCR2 acggcgaaatgcgtcc-ccaactccatcgtcatg--------------------------

VeANS_PCR2 acggcgaaatgcgcccgcaaactccactcacatg--------------------------

*************.** * *******.. ****

VaANS_WGS gagcaacggcaagtacaagagtattcttcacagggggcttgtcaacaaggagaaggtgag

VaANS_PCR1 gagcaacggcaagtacaagagtattcttcacagggggcttgtcaacaaggagaaggtgag

VaANS_PCR2 ------------------------------------------------------------

VeANS_PCR2 ------------------------------------------------------------

VaANS_WGS gatctcgtgggcggttttctgtgagcctcccaaggagaagatcatcctgaagccgctgcc

VaANS_PCR1 gatctcgtgggcggttttctgtgagcctcccaaggagaagatcatcctgaagccgctgcc

VaANS_PCR2 ------------------------------------------------------------

VeANS_PCR2 ------------------------------------------------------------

VaANS_WGS ggaaaccgtctccgaggagaagcctgccatctttccgcctcggactttctctgagcatat

VaANS_PCR1 ggaaaccgtctccgaggagaagcctgccatctttccgcctcggactttctctgagcatat

VaANS_PCR2 ------------------------------------------------------------

VeANS_PCR2 ------------------------------------------------------------

VaANS_WGS ccagcacaagctgttcaggaagagtcaggagaatctcctctctactgaagacgctgctct

VaANS_PCR1 ccagcacaagctgttcaggaagagtcaggagaatctcctctctactgaagacgctgctct

VaANS_PCR2 ------------------------------------------------------------

VeANS_PCR2 ------------------------------------------------------------

VaANS_WGS caaaagtactaaagaagctgctctcatctctactgatgaagctgcgctcatctctactga

VaANS_PCR1 caaaagtactaaagaagctgctctcatctctactgatgaagctgcgctcatctctactga

VaANS_PCR2 ------------------------------------------------------------

VeANS_PCR2 ------------------------------------------------------------

VaANS_WGS agaagctgctatcatctctactaatggagctgatctcatctctactaaagaagctgcttt

VaANS_PCR1 agaagctgctatcatctctactaatggagctgatctcatctctactaaagaagctgcttt

VaANS_PCR2 ------------------------------------------------------------

VeANS_PCR2 ------------------------------------------------------------

VaANS_WGS catctctcctaacggagctgctctcatctctacataa----------------------

VaANS_PCR1 catctctcctaacggagctgctctcatctctacataagatgctgcttaattaatggagc

VaANS_PCR2 -----------------------------------------------------------

VeANS_PCR2 -----------------------------------------------------------
